# Rising global burden of migraine among adolescents and young adults: a 30-year analysis (1990–2021)

**DOI:** 10.3389/fneur.2025.1652468

**Published:** 2025-09-01

**Authors:** YanPi Li, NaiChong Hu, BiFa Fan, XiYun Wang, Peng Mao, Yi Zhang, YiFan Li

**Affiliations:** ^1^Graduate School, Beijing University of Chinese Medicine, Beijing, China; ^2^Department of Pain Management, China-Japan Friendship Hospital, Beijing, China

**Keywords:** adolescent, young adult, epidemiology, Global Burden of Disease, migraine disorders

## Abstract

**Background:**

Migraine significantly impacts the physical and mental health of adolescents and young adults (AYA, aged 10–24 years). This study aims to assess global trends in migraine incidence, prevalence, and DALYs in this age group from 1990 to 2021, providing evidence to guide prevention and policy efforts.

**Materials and methods:**

Data were obtained from the Global Burden of Disease (GBD) 2021 study, encompassing AYA’s migraine burden across 204 countries and territories over the past 30 years, stratified by sex, age, socio-demographic index (SDI), location and year. The assessment analyzed incidence, prevalence, and disability-adjusted life years (DALYs).

**Results:**

Between 1990 and 2021, the global burden of migraine among AYA increased markedly in terms of absolute case numbers. Incident cases rose by 23.50%, prevalent cases by 24.82%, and DALYs by 24.94%. Despite these increases, the overall rates and age-standardized rates (ASRs) remained relatively stable, suggesting that population growth and aging are key drivers. The burden was consistently higher in females and in high-SDI regions; however, the rate of increase was greater in males, gradually narrowing the sex gap. Age-wise, those aged 10–14 had the highest incidence rate (45.9%), while the 20–24 group bore the greatest prevalence (39.8%) and DALY burden (39.9%). Among 21 regions, Western Europe recorded the highest ASIR (2272.50 per 100,000), while Tropical Latin America had the highest ASPR (27542.29 per 100,000) and ASDR (1011.78 per 100,000). Nationally, Belgium had the highest ASIR (2758.02 per 100,000), and Brazil had the highest ASPR (27592.69 per 100,000) and ASDR (1013.43 per 100,000). However, projections indicated that ASIR, ASPR, and ASDR will continue to rise by 2035.

**Conclusion:**

Global migraine burden surged (1990–2021), with high-SDI regions facing highest DALYs and low-SDI areas underdiagnosed. AYA (10–14, especially females) show peak incidence due to hormonal/social factors. Despite projected ASR decline, cases will rise, demanding precision interventions: healthcare access, sex-specific strategies, and school-based programs. Urgent global efforts are required to promote equitable access to migraine care and prevention, as well as to advance research on emerging risk factors, such as air pollution, prolonged screen exposure, chronic stress, academic pressure, and others.

## 1 Introduction

Migraine is a chronic neurovascular disorder characterized by recurrent moderate to severe unilateral headaches, often accompanied by nausea, photophobia, and phonophobia (1–3). The pathogenesis involves complex pathophysiological mechanisms, including both central and peripheral pathways—such as peripheral and central sensitization, lack of habituation, thalamocortical dysrhythmia, and motor cortex hyperexcitability (4). Based on the presence or absence of aura preceding the headache and associated symptoms, migraine can be categorized into two primary types: migraine without aura (MO) and migraine with aura (MA) (2).

It is a complex genetic condition with intricate pathogenic mechanisms and varied clinical manifestations (5). As the foremost cause of disability worldwide for individuals under 50 years of age, migraine’s prevalence is on the rise (1). According to the latest Global Burden of Disease (GBD) 2021 data, approximately 1.16 billion people are affected by migraine, imposing substantial individual and socioeconomic burdens (6, 7). Notably, adolescents and young adults have shown a rapid increase in migraine incidence rates (6, 8–10). Despite the substantial contribution of migraine to DALYs in AYA, its implications for global health policy remain insufficiently addressed (11).

According to the WHO, adolescents and young adults (AYA) encompass both adolescence (10–19 years), including early (10–14 years) and late adolescence (15–19 years) subgroups, and young adulthood (20–24 years) (12). A GBD 2019 study revealed that headache disorders constitute the leading cause of disability-adjusted life years (DALYs) among the 10–24 age group, with particularly high prevalence of migraine in this population. However, current global health initiatives demonstrate insufficient attention to headache and migraine disorders in this demographic cohort (11). Compared to adults and the elderly, migraine in AYA presents even greater challenges, significantly affecting their quality of life. This includes notable social limitations, reduced physical activity, and increased school absenteeism, which can contribute to higher dropout rates (10, 13, 14). Additionally, migraine in this age group is associated with several comorbidities, such as asthma, allergies, sleep disturbances, and emotional or behavioral issues like depression and anxiety (15). Furthermore, early-onset migraine increases the risk of physical and psychological comorbidities in adulthood, potentially leading to long-term adverse outcomes (16, 17). The adolescent brain remains under development, particularly in the prefrontal cortex which governs impulse control and executive decision-making, rendering this population neurobiologically vulnerable to substance dependence (18). Medication overuse in this developmental period may induce secondary migraine disorders (19), establishing a detrimental cyclical pattern. Critically, adolescent substance use disorders demonstrate significant longitudinal stability into adulthood (20), with severe consequences including functional impairment, premature mortality, and increased propensity for violent behaviors (21).

Understanding the long-term epidemiological patterns of migraine in AYA is essential to inform timely and effective public health responses (22). Analyzing the period from 1990 to 2021 offers a critical longitudinal perspective, capturing three decades of changes across demographic, healthcare, and sociopolitical contexts. This 30-year span enables robust trend detection, intergenerational comparisons, and evaluation of progress—or stagnation—in migraine burden reduction among young populations. It also establishes a baseline for assessing recent disruptions, notably the COVID-19 pandemic, which has altered lifestyles, mental health, and healthcare utilization—factors known to influence headache disorders. Ultimately, this research aims to elucidate the long-term epidemiological patterns of migraine in adolescents and young adults (AYA), facilitate the implementation of targeted public health interventions, and advance technological innovations in understanding the pathogenesis, prevention, clinical management, and therapeutic approaches for migraine in this population.

Only two pivotal studies have investigated migraine burden among adolescents and young adults (10–24 years) in the past 5 years, with both limited to data up to 2019. The study by Ge et al. (23) specifically compared migraine burden with tension-type headache burden in this age group, while Yang and Cao (8) examined differential burden patterns across age strata, geographical regions, and socio-demographic index (SDI) levels. However, these findings have become potentially outdated and inadequate for addressing current needs. To bridge these critical gaps, our study leverages the most recent 2021 Global Burden of Disease (GBD) dataset to comprehensively update burden trends according to age, region, and SDI classification. We further extend prior research by incorporating novel gender-disaggregated analyses presented through intuitive lollipop chart visualizations, applying advanced frontier analysis to quantify SDI-specific effects on migraine burden, and most importantly, generating pioneering 10-year global projections of migraine burden trajectories.

## 2 Methods

The data on migraine in AYA aged 10–24 years for this study were sourced from GBD 2021, accessed from December 1, 2024, to February 28, 2025. This is a publicly available, anonymized dataset, and no personally identifiable information was available or accessible at any stage of the research. The dataset provides the latest estimates of epidemiological data on the burden of 371 diseases and injuries across 21 GBD regions and 204 countries and territories from 1990 to 2021 (22, 24, 25). The GBD database, led by the Institute for Health Metrics and Evaluation (IHME) at the University of Washington, integrates data from diverse sources and utilizes advanced analytical methods, including the Cause of Death Ensemble model, spatiotemporal Gaussian process regression, and Bayesian meta-regression tool DisMod-MR 2.1, to assess both the epidemiology and the non-fatal burden of migraine globally (11, 26–28). Further details on the GBD 2021 methodology can be found in other publications (25, 29–31). Data for this study were extracted from the Global Health Data Exchange (GHDx) Results Tool^1^ (32). All analytical procedures adhered to the Guidelines for Accurate and Transparent Health Estimates Reporting (GATHER), with the completed checklist provided in Supplementary material (32, 33).

### 2.1 Disease definition

In GBD 2021, migraine is classified as a primary headache disorder, falling under the broader categories of neurological disorders (second level) and non-communicable diseases (first level). Defined by the International Classification of Headache Disorders, 3rd edition (ICHD-3), migraine is characterized by recurrent, moderate to severe, unilateral pulsating head pain (8, 19). Consistent with most epidemiological studies, GBD 2021 does not distinguish between migraine with and without aura, instead focusing on the overall burden of migraine (3). In previous versions of the GBD, medication-overuse headache (MOH) was classified as a separate entity. However, in GBD 2021, MOH has been reclassified and removed as a distinct category, with approximately 73% of MOH DALYs now attributed to migraine-induced MOH (34). According to the International Classification of Diseases (ICD), migraine is coded 346–346.93 in the 9th edition and G43–G43.919 in the 10th edition (25).

### 2.2 Socio-demographic index

The SDI, introduced in GBD study, is a composite measure that quantifies a country’s level of development based on three key factors: fertility rate (total fertility rate under 25 years), education (mean years of schooling for individuals aged 15 and older), and income (lag-distributed income per capita, adjusted for time). The SDI ranges from 0 to 1, with higher values indicating greater socioeconomic development. Based on the 2021 SDI values, countries are grouped into five quintiles: low, low-middle, middle, high-middle, and high (35). The SDI is used to assess the relationship between sociocultural and macroeconomic factors and health outcomes, with higher SDI values typically associated with lower disease incidence and mortality rates.

### 2.3 Statistical analyses

The following software was used for the analyses: R (version 4.4.2), Joinpoint Regression Program (version 5.0.2), and JD_GBDR (V2.35.1, Jingding Medical Technology Co., Ltd.). For trend analysis, statistical significance was defined as a two-sided *p*-value of <0.05.

### 2.4 Preliminary analysis

This study examines the spatiotemporal trends in the number of migraine cases, crude rates, and age-standardized rates (ASR) of incidence, prevalence, and DALYs for AYA from 1990 to 2021, including the development of regression models and visualizations of these trends. Stratified comparisons were made by sex, age group (10–14, 15–19, 20–24 years), SDI, location (global, 21 GBD regions, 204 countries), and year. Each rate is reported per 100,000 population with 95% uncertainty intervals (UI), calculated from 1,000 iterations of sampling, with the upper and lower bounds derived from the 2.5th and 97.5th percentiles of the uncertainty distribution (11, 36–38). DALYs represent the disease burden, calculated as the sum of years of life lost (YLLs) due to premature mortality and years lived with disability (YLDs) (39). Since the GBD does not directly attribute deaths to headache disorders, the DALYs for headaches are effectively equivalent to the YLDs (40). ASR were used to eliminate the effects of population structure differences.

DALYs=YLLs+YLDs

The EAPC was calculated from the slope of the regression line. A positive EAPC with the lower bound of the 95% confidence interval (CI) >0 indicates an increasing trend, while a negative EAPC with the upper bound of the 95% CI <0 reflects a decreasing trend (26). A 95% CI with both upper and lower bounds spanning 0 suggests a stable trend. The formula applied was as follows:

EAPC=100×(exp(β)−1)

where *β* is the estimated slope from the regression model.

Total percentage change (PC) is used to measure the overall change in incidence, prevalence and DALY rates of AYA migraines from 1990 to 2021. The calculation formula is as follows:

$$\text{Total percentage change}=(\frac{2021\;\text{value}-1990\;\text{value}}{1990\;\text{value}})*100\%$$

All PC values used in this paper were directly downloaded from the GBD database and retained to two decimal places.

### 2.5 Advanced analysis

#### 2.5.1 Joinpoint regression model

Joinpoint regression analysis was employed to evaluate temporal trends in the age-standardized incidence rate (ASIR) (8). This method identifies significant inflection points where trend patterns shift, thereby segmenting the overall trend into distinct periods (41, 42). For each segment, the annual percentage change (APC) and its 95% uncertainty interval (UI) were calculated to quantify temporal trends. To ensure the robustness of the analysis, the Monte Carlo permutation method was applied, generating 4,499 randomly permuted datasets, with Bonferroni correction applied to account for multiple comparisons and maintain the overall significance level (43). Trends were classified as follows: an increasing trend was defined when both the APC estimate and the lower boundary of its 95% UI exceeded zero, while a decreasing trend was indicated when both the estimate and the upper boundary of its 95% UI were below zero. If neither condition was met, the trend was considered stable. To summarize trends over the entire study period (1990–2021), the average annual percent change (AAPC) was calculated (37, 41).

#### 2.5.2 Cross-country inequality analysis

The slope index of inequality (SII) and concentration index are widely used to measure the unequal distribution of the migraine burden among AYA in various countries. The SII assesses absolute inequality, ranks countries or regions based on the SDI and conducts a robust weighted regression of the DALY rate based on these rankings (44). The concentration index assesses relative inequality by numerically integrating the area under the Lorenz concentration curve (45). A positive concentration index value indicates that DALYs are concentrated in countries with a higher SDI, while a negative concentration index value indicates that DALYs are more prevalent in countries with a lower SDI (44).

#### 2.5.3 Bayesian age-period-cohort model analysis

We used the Bayesian age-period-cohort (BAPC) model to predict development trends in migraine burden over the coming decades. The BAPC model estimates the posterior distribution by integrating prior information with sample data, enabling inference of unknown parameters. The commonly used algorithm is the integrated nested Laplace approximation (INLA) (43).

#### 2.5.4 Frontier analysis

Frontier analysis is a statistical method used to identify countries or regions with the lowest disease burden under a given SDI. These countries or regions are regarded as the “frontier” and drive the boundary. The “effective difference” represents the distance between the disease burden of a specific country or region and the frontier. It indicates the gap between the actual situation observed in a country or region given its SDI and what can be achieved (46).

## 3 Results

### 3.1 Global AYA migraine burden

From 1990 to 2021, there was an increase in the number of incident cases, prevalent cases, and DALYs of migraine among AYA globally (Supplementary Table 1). Conversely, the ASIR (EAPC = 0.05, 95% CI: −0.39 to 0.50), age-standardized prevalence rate (ASPR, APC = 0.11, 95% CI: −0.35 to 0.57) and age-standardized DALY rate (ASDR, EAPC = 0.11, 95% CI: −0.33 to 0.56) remained relatively stable over the same period.

Specifically, the incident cases increased by 23.50%, rising from 27.87 million (95% UI: 22.05 to 34.08) in 1990 to 34.42 million (95% UI: 27.31 to 42.20) in 2021. Similarly, the prevalent cases increased by 24.82%, rising from 243.62 million (95% UI: 190.06 to 304.40) in 1990 to 304.08 million (95% UI: 235.57 to 379.32) in 2021. Meanwhile, the DALYs increased by 24.94%, rising from 8.98 million (95% UI: 0.72 to 21.85) in 1990 to 11.22 million (95% UI: 0.87 to 27.28) in 2021.

From 1990 to 2021, the trends in incidence, prevalence, and DALY rates remained relatively stable, with a slight increase in 2021 compared to 1990. The AAPC ranged from 0.02 to 0.1, with *p* < 0.05.

### 3.2 Global trends by sex

**Figure 1:**
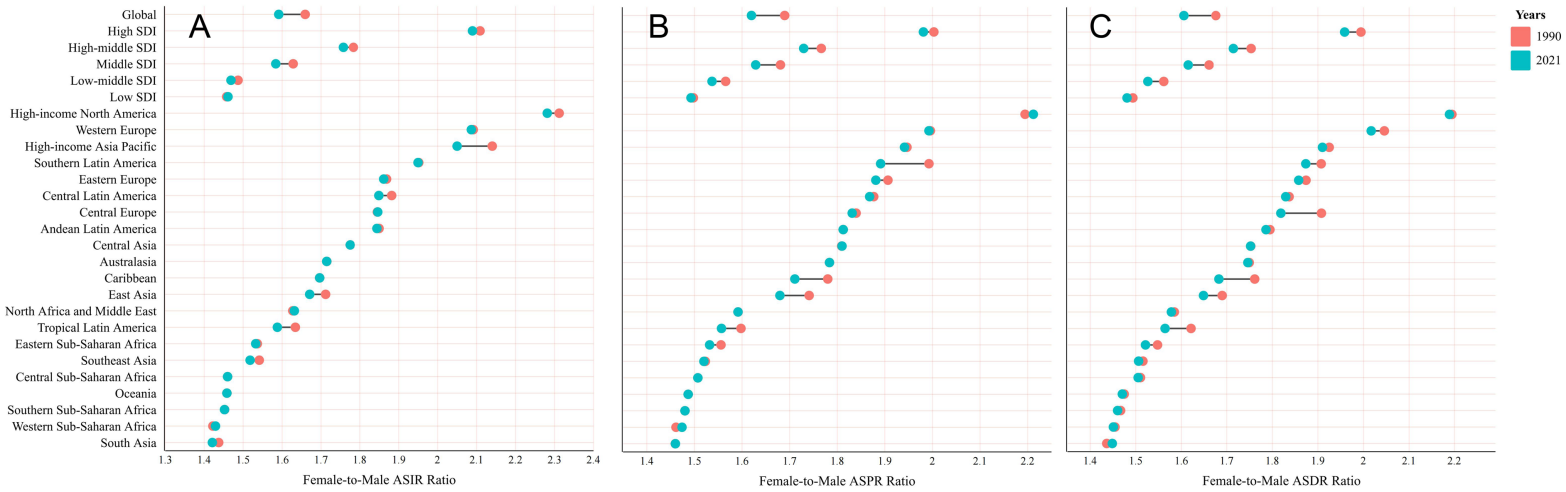
Female-to-male ratios of ASR for migraine in 1990 and 2021. **(A)** ASIR; **(B)** ASPR; **(C)** ASDR.

From 1990 to 2021, the global burden of migraine in female has consistently been higher than in male, with this sex disparity becoming more pronounced in regions with higher SDI levels (Figure 1). Among AYA, female consistently exhibited significantly higher rates than male in terms of migraine incidence, prevalence, and DALY (Supplementary Figure 1). During this period, the sex gap narrowed in most regions, except in low SDI regions, North Africa and the Middle East, Western Sub-Saharan Africa, and High-Income North America (Figure 1). In 1990, the ASDR for female was 1.68 times higher than that for male globally, whereas in 2021, this ratio decreased to 1.61 (Figure 1 and Supplementary Table 2). In 2021, at high SDI level, the ASDR for female was 1.96 times that for male, compared to a ratio of 1.48 in low SDI regions (Figure 1 and Supplementary Table 2).

**Figure 2:**
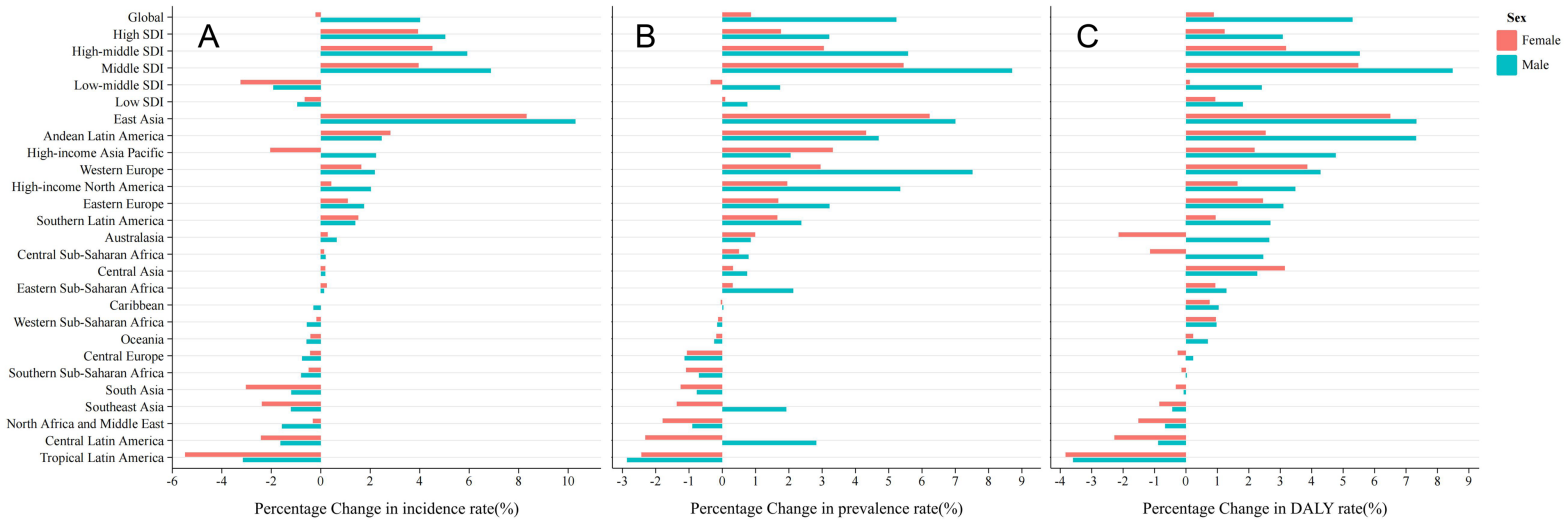
Percentage change rate by sex from 1990 to 2021. **(A)** Incidence Rate; **(B)** Prevalence Rate; **(C)** DALY Rate.

Globally, from 1990 to 2021, both the prevalence and DALY rates for migraine have shown an increasing trend, with male experiencing a larger increase than female (Figure 2 and Supplementary Table 3). The PC in prevalence and DALY rates for male is approximately 6 times greater than that for female. As for the incidence rate, there is a slight decline in female, with a 0.21% decrease in 2021 compared to 1990, while male have seen an increasing trend, with a 4.02% rise since 1990.

Among female, the ASIR and ASPR of migraine exhibited minimal changes over the period from 1990 to 2021 (Supplementary Table 1). Specifically in global, the ASIR slightly decreased from 2270.98 per 100,000 (95% UI: 1503.99 to 3180.91) in 1990 to 2258.91 per 100,000 (95% UI: 1496.50 to 3178.99) in 2021, with an AAPC of −0.02% (95% UI: −0.03 to −0.01). In contrast, the ASPR increased marginally from 19764.45 per 100,000 (95% UI: 15013.87 to 25153.75) to 19982.87 per 100,000 (95% UI: 15191.63 to 25518.51), reflecting an EAPC of 0.05 (95% UI: 0.04 to 0.06). Similarly, the ASDR showed a slight increase, rising from 725.79 per 100,000 (95% UI: 55.16 to 1766.55) in 1990 to 734.07 per 100,000 (95% UI: 56.48 to 1784.99) in 2021, with an EAPC of 0.06 (95% UI: 0.05 to 0.07).

Whereas, among male, there was a more pronounced increase in migraine burden (Supplementary Table 1). The ASIR rose from 1368.65 per 100,000 (95% UI: 913.06 to 1926.31) in 1990 to 1419.46 per 100,000 (95% UI: 946.54 to 1993.82) in 2021, with an AAPC of 0.12% (95% UI: 0.11 to 0.13). The ASPR for male also increased, from 11698.38 per 100,000 (95% UI: 8807.39 to 15027.08) to 12336.87 per 100,000 (95% UI: 9243.96 to 15823.68), reflecting an EAPC of 0.18 (95% UI: 0.17 to 0.19). The ASDR for male showed a similar trend, rising from 434.10 per 100,000 (95% UI: 38.40 to 1054.70) in 1990 to 458.21 per 100,000 (95% UI: 38.15 to 1116.50) in 2021, with an EAPC of 0.18 (95% UI: 0.18 to 0.19).

### 3.3 Global trends by age groups

**Figure 3:**
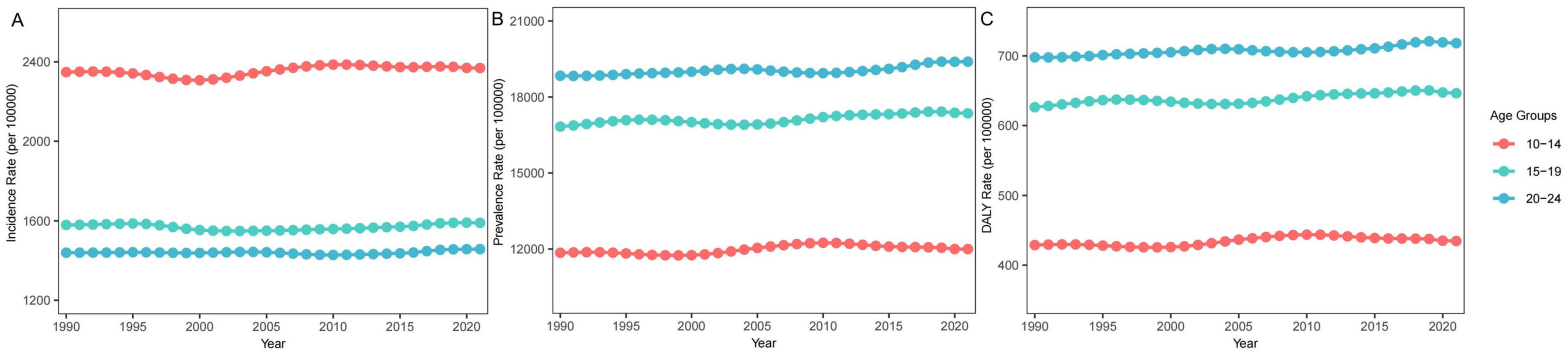
Trends in migraine burden by age group from 1990 to 2021. **(A)** Incidence Rate; **(B)** Prevalence Rate; **(C)** DALY Rate.

Compared to 1990, the age distribution of migraine burden remained relatively stable globally and across most regions in 2021 (Figure 3; Supplementary Figure 2). From 1990 to 2021, adolescents aged 10–14 consistently exhibited the highest incidence rate of migraine, accounting for 43.7% of the global migraine incidence rate among AYA in both 1990 and 2021 (Supplementary Figure 2). The incidence rate decreased with increasing age. In 2021, the 10–14 age group represented 45.9%, or 15.8 million, of the 34.4 million new migraine cases among AYA (Supplementary Figure 2A). Conversely, the prevalence and DALY rates were highest in the 20–24 age group, with a decrease as age decline. In 2021, globally, young adults aged 20–24 accounted for 39.8% of the prevalence rate and 39.9% of the DALY rate among individuals aged 10–24 (Supplementary Figures 2D,F).

### 3.4 Global trends by SDI quintiles

**Figure 4:**
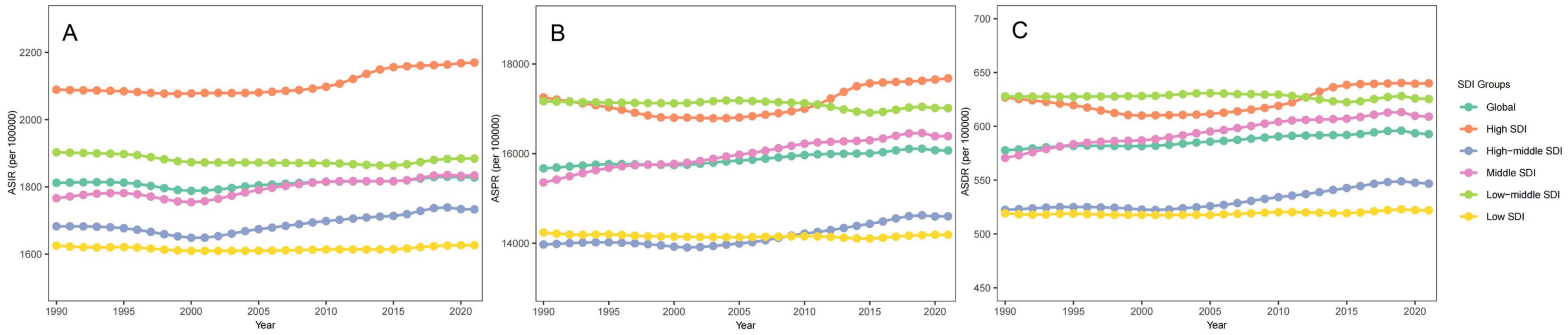
Trends in ASR of migraine burden from 1990 to 2021. **(A)** ASIR; **(B)** ASPR; **(C)** ASDR.

Between 1990 and 2021, the migraine landscape across various SDI regions showed a multifaceted shift in its burden (Figure 4 and Supplementary Table 1). The prevalent and incident cases of migraine decreased in high and high-middle SDI regions, whereas both metrics increased in the middle, low-middle, and low SDI regions (Supplementary Table 1). The ASIR increased slightly in all regions except the low-middle SDI region, where it decreased with an AAPC of −0.03% (95% UI: −0.04 to −0.03). In the high SDI region, the ASIR of migraine increased most rapidly, with an AAPC of 0.12% (95% UI: 0.10 to 0.14).

In 1990, the high SDI region recorded the highest ASIR and ASPR, while the low-middle SDI region had the highest ASDR (Figure 4). In contrast, the low SDI region had the lowest ASIR and ASDR, while the high-middle SDI region reported the lowest ASPR. By 2021, the High SDI region exhibited the highest ASIR, ASPR, and ASDR, highlighting its significant burden. Conversely, the Low SDI region had the lowest values across all three metrics.

### 3.5 Regional and national trends

**Figure 5:**
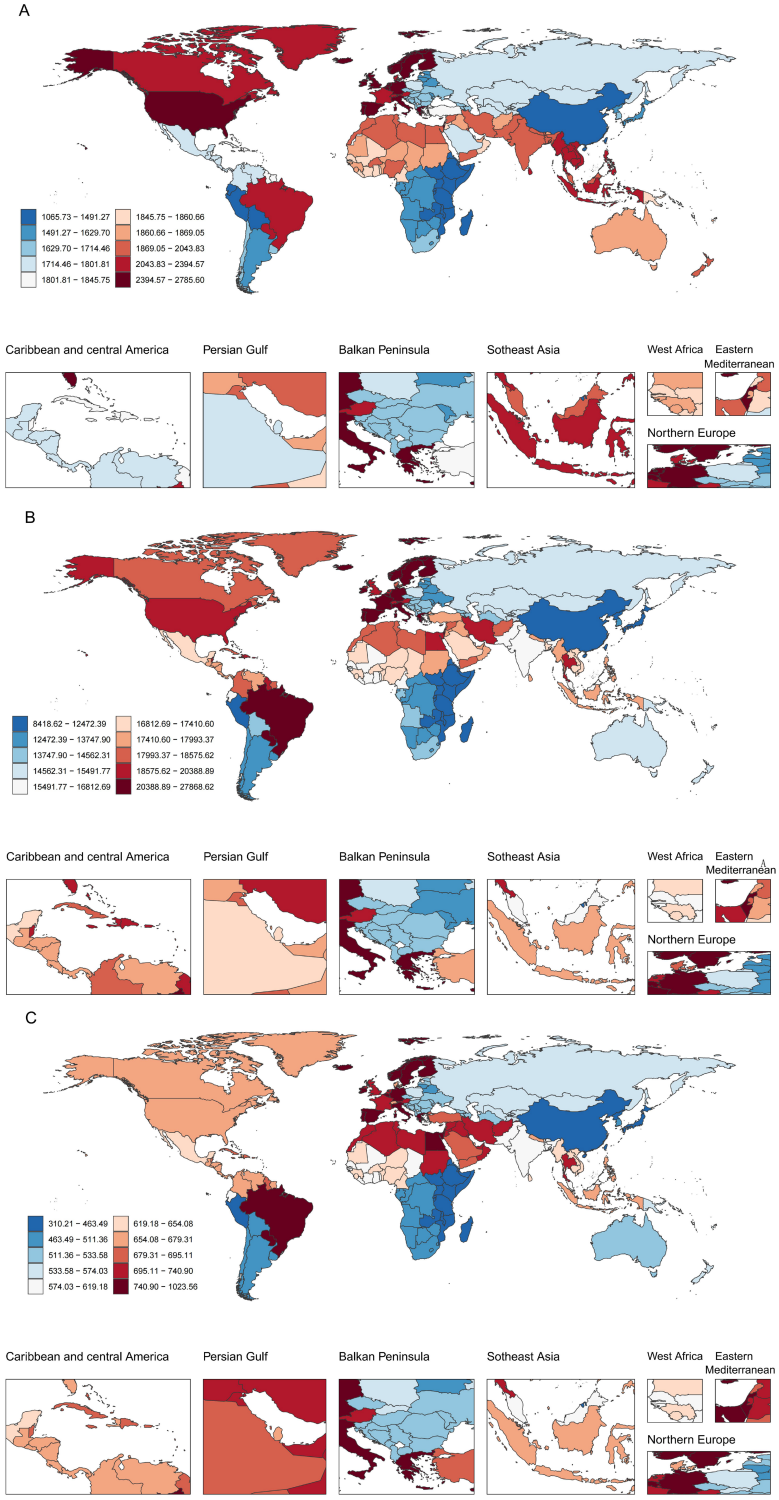
Global distribution of ASR of migraine burden among AYA in 2021. **(A)** ASIR; **(B)** ASPR; **(C)** ASDR.

The absolute number of prevalent, incident and DALY cases associated with migraine among AYA has increased over time in most regions (Figure 5 and Supplementary Tables 1, 4). In 2021, India recorded the highest incident cases (7.54 million), followed by China (3.50 million) and Nigeria (1.57 million), which collectively accounted for nearly 37% of global incident cases (Supplementary Table 4). In terms of prevalent and DALY cases, India also ranks the first, followed by China and Brazil (Supplementary Table 4).

From 1990 to 2021, the ASPR, ASIR and ASDR have fluctuated across regions, with overall trends remaining relatively stable (Supplementary Table 1). Among these regions, East Asia, Andean Latin America and Tropical Latin America exhibited upward trends. In contrast, regions such as high-income Asia Pacific, South Asia and Southeast Asia tend to decline. In 2021, Western Europe had the highest ASIR, while Tropical Latin America had the highest ASPR and ASDR.

At the national level, Norway and Singapore have the most obvious upward trends in ASPR, ASIR and ASDR, while Thailand and the Republic of Korea showed declining trends (Supplementary Table 4). The top three countries with the highest ASIR are Belgium, Italy and Norway, all of which are European countries; while Brazil, Paraguay and Belgium have relatively high ASPR and ASDR.

### 3.6 Joinpoint regression model

**Figure 6:**
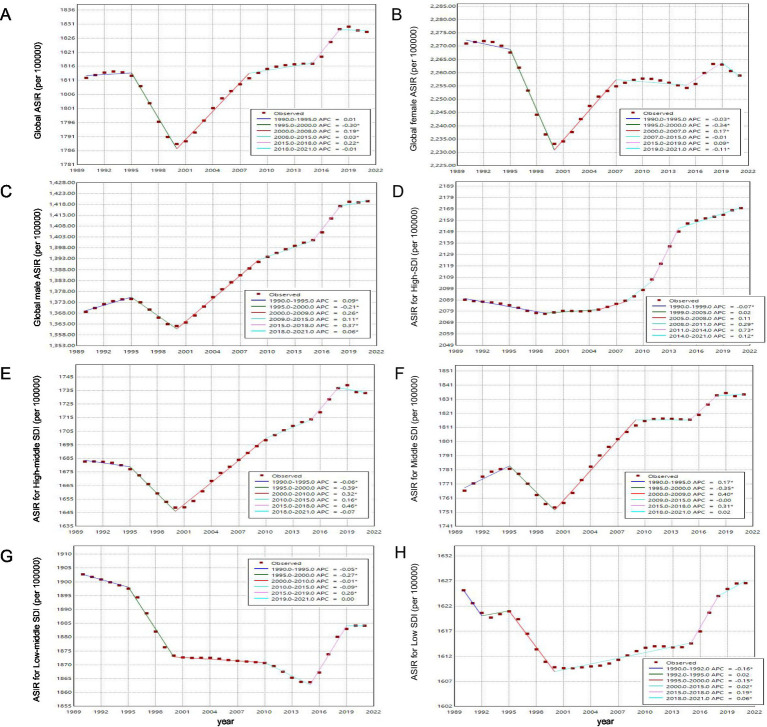
Global trends in ASIR of migraine among AYA from 1990 to 2021. Asterisks (*) indicate that the APC is significantly different from zero at the alpha = 0.05 level.

The results of the joinpoint regression analyses are shown in Figure 6. Globally, the ASIR exhibited a fluctuating pattern: (1990–1995: increased by 1%; 1995–2000: declined by 30%, *p* < 0.05; 2000–2008: increased by 19%, *p* < 0.05; 2008–2015: increased by 3%, *p* < 0.05; 2015–2018: increased by 22%, *p* < 0.05; 2018–2021: declined by 1%). This pattern was mirrored in male globally. For female globally, the trend exhibited similar inflection point during 1995–2000, with a more pronounced decline of 34% (*p* < 0.05). In high SDI, the fastest growth in ASIR occurred between 2011 and 2014, surging by 73% (*p* < 0.05). In middle SDI, the most rapid increase was observed between 2000 and 2009, with an increment of 40% (*p* < 0.05). In contrast, in high-middle, low-middle, and low SDI regions, the steepest increase in ASIR was noted around 2015–2018, rising by 46, 28, and19%, respectively (all *p*-values <0.05).

### 3.7 Cross-country inequality analysis

**Figure 7:**
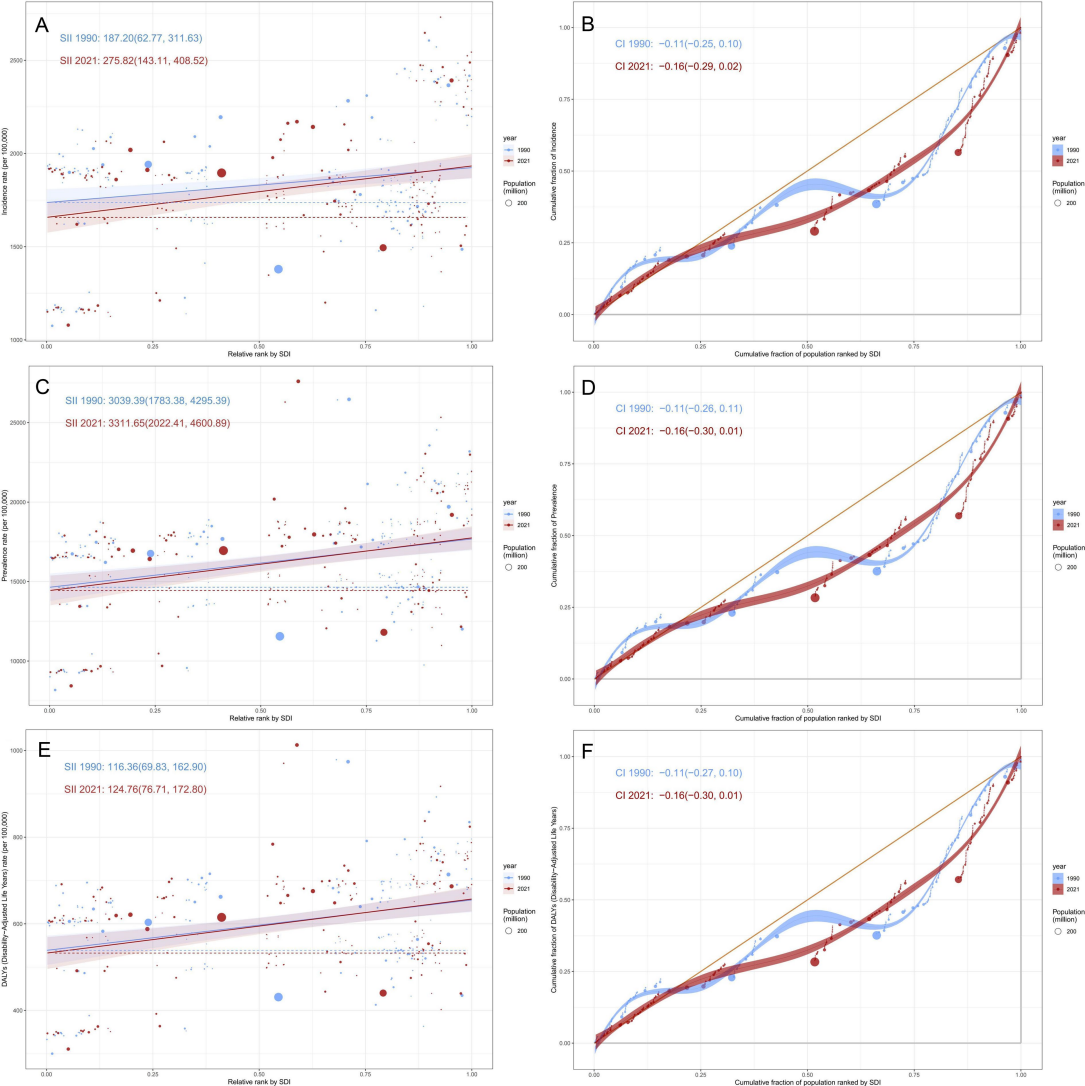
SDI-related health inequality regression and concentration curves for the ASR of migraine among AYA from 1990 to 2021. **(A)** SII of incidence rate; **(B)** Concentration index of incidence rate; **(C)** SII of prevalence rate; **(D)** Concentration index of prevalence rate; **(E)** SII of DALY rate; **(F)** Concentration index of DALY rate.

We identified significant absolute and relative inequalities in migraine burden among countries with different SDI (see Figure 7). In countries with a higher SDI level, the DALY rate is higher and more disproportionately concentrated. In 1990, the SII for prevalence was 3039.39, indicating that the prevalence rate in the highest SDI country was 3039.39 per 100,000 population higher than in the lowest SDI country. This gap further widened to 124.755 in 2021. The trends of incidence and prevalence rates are similar. Meanwhile, the concentration index showed no clear direction, indicating that the disease burden did not significantly lean toward countries with either high or low SDI.

### 3.8 Global disease burden prediction for migraine in AYA to 2035

**Figure 8:**
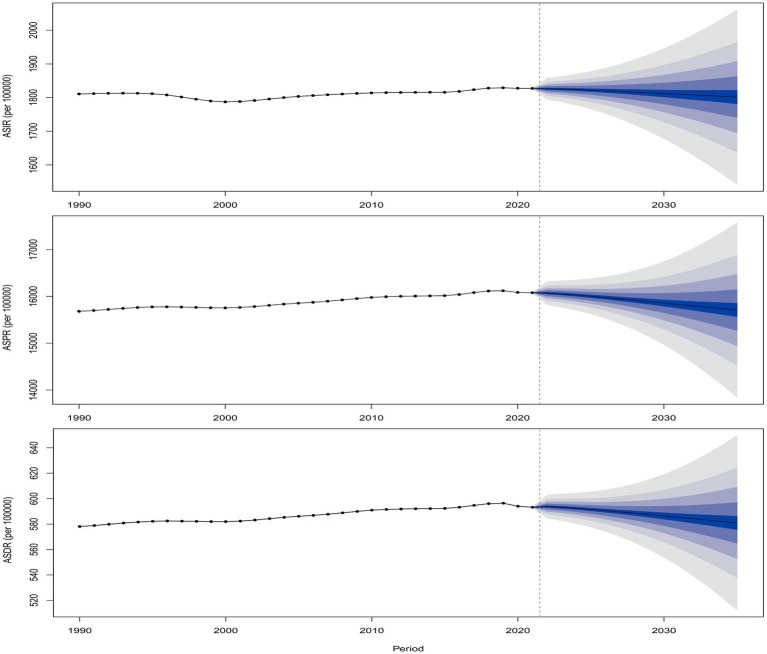
Global predicted trends in ASR of migraine from 2022 to 2035. **(A)** ASIR; **(B)** ASPR; **(C)** ASDR.

The projected counts and ASR of incidence, prevalence, and DALY for migraine through 2035 are illustrated in Figure 8; Supplementary Figure 3. Globally, the ASIR, ASPR, and ASDR of migraine are predicted to increase until 2035, while the absolute number of incident cases, prevalent cases, and DALYs are expected to rise initially and then decline.

### 3.9 Frontier analysis

The frontier analysis, represented by a solid black line indicating the minimum achievable ASDR across SDI levels (Supplementary Figure 4), revealed significant global disparities in migraine burden. We quantified these disparities by calculating the effective difference—the distance between observed DALY rates and the frontier line—for each region, reflecting the potential for burden reduction. Analysis showed increasing effective differences and their variability with higher SDI levels. In 2021, 15 countries with the greatest potential for burden reduction were identified: Brazil, Paraguay, Belgium, Italy, Germany, Egypt, Spain, Greece, Norway, Israel, Iceland, Sweden, Malta, Finland, and Monaco.

## 4 Discussion

Migraine is a prevalent neurological disorder and a leading cause of disability in AYA, often underdiagnosed and undertreated, impacting quality of life, including family, leisure, and educational or occupational activities (47, 48). Furthermore, migraines in AYA can persist or worsen into adulthood, potentially evolving into chronic, refractory conditions that impose a lasting burden (8, 10). This study provides a comprehensive and updated analysis of global migraine incidence, prevalence, and DALYs among AYA from 1990 to 2021, complemented by advanced statistical evaluations of trends, inequality, and predictions, stratified by age and sex. These studies can reveal the management status of migraine across different countries and regions worldwide, in various age groups and time periods, evaluate the effectiveness and shortcomings of current management measures, and provide a scientific basis for the development of targeted policies in the future.

Between 1990 and 2021, the global burden of migraine among AYA increased significantly, with the number of incident cases rising by 23.50%, prevalent cases by 24.82%, and DALYs by 24.94%, respectively. Despite these increases, the ASR have remained relatively stable, suggesting that the underlying causes of migraine have persisted and that current treatments have not significantly reduced the overall burden. This may be due to limited accessibility to effective therapies and a lack of robust longitudinal data on treatment outcomes (40). The increase in DALYs highlights the growing economic and societal impact of migraine, particularly in terms of workforce productivity loss. While significant advancements have been made in migraine diagnosis and treatment over the past three decades, societal risk factors such as unhealthy lifestyles, physical inactivity, prolonged screen time, academic and occupational stress, and environmental pollution have concurrently increased (36, 49–51), offsetting potential improvements in disease burden. To address these challenges, targeted interventions are urgently needed. Investments in longitudinal studies to better understand region-specific risk factors and treatment outcomes are critical for developing tailored, precision-based policies. Additionally, improving access to effective therapies and enhancing public health initiatives to mitigate modifiable risk factors could significantly reduce the global burden of migraine.

In 2021, migraine affected approximately 304.08 million AYA globally, with 34.42 million new cases and an estimated 11.22 million DALYs lost. Notably, the highest ASPR and ASDR were observed in Tropical Latin America, while Western Europe recorded the highest ASIR. These regional disparities likely arise from a complex interplay of genetic, environmental, and socioeconomic factors. In Tropical Latin America, elevated temperatures may contribute to the increased migraine burden (52). Studies indicate that worsening climate conditions, including rising temperatures, extreme weather events, and increasing pollution, are associated with increased migraine severity, duration, and frequency (53). Additionally, thermal stress can trigger migraine attacks by promoting vasodilation in small arteries during thermoregulatory responses (54). Moreover, limited access to healthcare, delayed diagnosis, and insufficient management exacerbate the disability burden in this region. The region’s relatively young demographic profile may also explain the higher disease burden among AYA (55). Conversely, Western Europe’s advanced healthcare infrastructure and diagnostic capabilities contribute to its high reported incidence. Lifestyle factors—including chronic stress, dietary patterns, and prolonged screen exposure—may further increase migraine susceptibility in this region. Several non-pharmacological interventions, such as manual therapy and exercise-based therapy, have demonstrated clinical efficacy in managing migraines (56). Implementing such cost-effective strategies in resource-limited settings could significantly alleviate the disease burden. Meanwhile, in high-income regions, reducing screen time and promoting extracurricular physical activities may serve as preventive strategies to curb rising migraine incidence among adolescents and young adults.

The BAPC model projects a slight decline in global ASIR, ASPR, and ASDR from 2022 to 2035 compared to 2021. However, the absolute number of cases for these metrics is expected to continue rising, indicating that the burden of migraine remains inadequately controlled and managed. This trend underscores a significant public health challenge. To address this, further research into the pathophysiology of migraine is imperative. Additionally, targeted, precision-based policies tailored to the specific burden trends of each country and region are essential to mitigate the growing impact of migraine.

The burden of migraine varies significantly across regions with different SDI levels, and these differences highlight the differentiated needs for global health policies. Cross-national inequality analysis shows that in higher SDI regions, the DALY rate is disproportionately concentrated, and inequality has intensified over time. In 2021, the ASIR, ASPR, and ASDR of migraine were highest in high-SDI regions, followed by low-middle-SDI, middle-SDI, and high-middle-SDI regions, while low-SDI regions recorded the lowest values. From 1990 to 2021, the burden of migraine (ASIR, ASPR, and ASDR) in high-SDI, high-middle-SDI, and middle-SDI regions showed a slight increase, whereas low-middle-SDI regions experienced a decline in ASIR, ASPR, and ASDR, and low-SDI regions saw a modest reduction in ASDR. These trends may be driven by multiple factors. In high-SDI regions, industrialization, urbanization, and modernization have introduced environmental and lifestyle changes, including increased exposure to environmental toxins, sedentary behaviors, and higher stress levels, which are known risk factors for migraine (1, 57). Additionally, migraine is a chronic and recurrent condition with no definitive cure, leading to a persistent demand for healthcare resources even in high-SDI settings (58).

In contrast, low-SDI regions exhibit a lower burden of migraine despite poorer healthcare standards. This phenomenon may be attributed to several factors. First, migraine-related research and reporting are less comprehensive in low-SDI regions, resulting in underdiagnosis and underreporting (59). Second, limited healthcare infrastructure and accessibility contribute to lower consultation and diagnosis rates, as well as poorer retention of medical records (23). Furthermore, lifestyle factors prevalent in low-SDI regions, such as lower levels of life stress, higher physical activity, and dietary differences, may play a protective role against migraine (49). Notably, as the SDI increases, the female-to-male ratio of migraine burden also rises, indicating that migraine remains predominantly a female-driven condition in higher-SDI regions. This highlights the urgent need for sex-specific approaches to migraine diagnosis and treatment. Despite these regional variations, the overall improvement in migraine burden from 1990 to 2021 has been suboptimal, suggesting that current treatment strategies remain inadequate. These findings underscore the critical need for further research breakthroughs in migraine management, particularly in understanding the environmental, genetic, and lifestyle factors that contribute to its burden. Future efforts should focus on integrating advanced diagnostic and therapeutic approaches from high-SDI regions into low-SDI settings, while addressing the unique healthcare challenges faced by female and underserved populations.

Joinpoint analysis revealed significant turning points in the global burden of migraine from 1990 to 2021. The decline in migraine prevalence from 1995 to 2000 may be attributed to the standardization of migraine diagnosis following the International Headache Society (IHS) diagnostic criteria, first issued in 1988 (19). These criteria provided a clear framework for early screening and diagnosis, which, coupled with increased research into migraine pathogenesis and treatment around 1990, led to the development and clinical application of more effective therapies. Concurrently, international efforts to improve child welfare, such as the establishment of the Children Rights International Network (CRIN) in 1995 and the promotion of international labor standards by UNICEF and the International Labour Organization in 1996, may have indirectly contributed to this decline (60). However, from 2000 to 2018, the ASIR of migraines gradually increased, potentially due to environmental factors such as climate change and atmospheric pressure fluctuations, which have been shown to trigger migraine attacks in some patients (61). Between 2019 and 2021, the global ASIR, ASPR and ASDR of migraine showed a declining trend, likely influenced by reduced diagnosis rates of non-COVID-19 conditions and changes in social behaviors during the pandemic (62, 63). These findings highlight the complex interplay of diagnostic advancements, environmental factors, and public health initiatives in shaping the temporal trends of migraine burden over the past three decades.

From 1990 to 2021, the burden of migraine in female remained consistently higher than in male, with relatively stable trends over time. This disparity is driven by a combination of biological, social, and healthcare access factors that disproportionately affect female (64–68). Pathophysiological mechanisms underlying migraine, including cortical spreading depression (CSD), trigeminovascular system activation, and neuroinflammation, exhibit heightened sensitivity in female due to the modulatory effects of estrogen (64, 67, 69). Estrogen influences pain pathways by altering the expression of neuropeptides such as calcitonin gene-related peptide (CGRP) and serotonin, which play critical roles in migraine pathogenesis (64). Additionally, fluctuations in estrogen levels during menstrual cycles, puberty, and other hormonal transitions further exacerbate migraine susceptibility in female (65). In contrast, the burden of migraine in male has shown a more pronounced increase in both incidence and prevalence, reflecting trends observed in adult populations. This rise may be attributed to improved healthcare access and awareness, leading to better recognition and diagnosis of migraine in male. Historically, cultural norms around masculinity and pain tolerance have contributed to underreporting and underdiagnosis in male, as men are often less likely to seek medical attention for pain-related conditions. However, shifting societal attitudes toward men’s health, coupled with increased focus on male migraine sufferers, have likely narrowed the sex gap in migraine recognition and treatment. For instance, public health campaigns and educational initiatives have raised awareness about migraine as a significant health issue for men, encouraging more male to seek diagnosis and treatment. This trend may also reflect the growing health challenges faced by male in modern society, including heightened work-related stress, greater physical labor demands, and limited awareness of personal health management. These factors are likely contributors to the rising incidence of migraine among male, mirroring trends observed in adult male (6). Consequently, future public health strategies should adopt a sex-balanced approach, with particular emphasis on prevention and early intervention for both male and female. Tailored interventions addressing the unique challenges faced by each sex—such as societal expectations and stress-related factors in male, and lower levels of physical activity in female—will be critical for improving outcomes.

Among AYA, global DALY and prevalence rates generally increased with age, while incidence rates decreased, peaking in the 10–14 age group. Notably, in regions such as Tropical Latin America, Andean Latin America, the Caribbean, and Central Latin America, adolescent incidence rates exceeded half those of the young adult population. This may be attributed to regional factors such as climate conditions, living environments, education levels, and health awareness (70). Adolescence represents a critical period of physiological and psychological development, during which hormonal changes, increased academic pressure, and the onset of puberty may heighten migraine susceptibility (71, 72). For female, the onset of menarche introduces additional hormonal fluctuations, further increasing migraine risk (73). These factors, combined with environmental and socioeconomic stressors, likely explain the elevated incidence rates observed in the 10–14 age group, particularly in high-risk regions. Addressing these challenges requires targeted interventions, including education on lifestyle factors (e.g., sleep hygiene, physical activity, and stress management) and the implementation of school-based health programs to promote early diagnosis and treatment. Such strategies are essential for reducing the burden of migraine in this vulnerable population and mitigating long-term health impacts.

## 5 Limitation

This study has several limitations that warrant consideration. First, challenges in migraine diagnosis, such as high misdiagnosis rates and variability in data collection across spatial and temporal contexts, may have introduced bias. The observed increase in migraine diagnosis rates in some regions may reflect improved health awareness and healthcare access rather than a true rise in disease prevalence. Second, as this study is based on data from the GBD database, which has inherent limitations. GBD relies primarily on secondary data sources, including national statistics, health surveys, and published literature, which vary in quality, completeness, and timeliness across regions (74). Additionally, the GBD applies modeling techniques to estimate data in regions lacking reliable sources (11). While this improves consistency, it may introduce bias if the assumptions do not reflect local disease patterns. These factors may lead to inaccuracies in estimating migraine burden, particularly in underrepresented populations. Third, the COVID-19 pandemic may have further compromised data accuracy, as healthcare systems prioritized pandemic response over non-urgent conditions, potentially leading to underdiagnosis and underreporting of migraine cases during this period. This highlights the need for standardized diagnostic criteria and robust data collection methods to distinguish between true increases in migraine incidence and improvements in case detection. Moreover, as our study primarily focuses on the adolescents and young adults, the findings may have limited generalizability to other age groups.

## 6 Conclusion

This study demonstrates a marked increase in the global number of incident cases, prevalent cases, and DALYs of migraine from 1990 to 2021, with pronounced disparities across SDI levels. High-SDI regions bear the greatest burden, driven by environmental, lifestyle, and diagnostic factors, while low-SDI regions face underdiagnosis and healthcare inequities. AYA aged 10–14, particularly female, exhibit disproportionately high incidence rates due to hormonal fluctuations, psychosocial stressors, and socioeconomic vulnerabilities. Projections suggest a modest decline in ASR by 2035, yet absolute case numbers will rise, emphasizing the urgency for precision-based interventions. Critical strategies include: (1) enhancing healthcare access in low-resource settings; (2) addressing sex-specific risks (e.g., physical inactivity in female, occupational stress in male); and (3) implementing school-based programs for AYA to promote early diagnosis and lifestyle modifications. Global collaboration is imperative to integrate advanced diagnostics, optimize resource allocation, and prioritize research on emerging risks, including climate change and digital health impacts. Concurrently, evidence-based self-management tools and population-level prevention campaigns targeting modifiable risk factors (e.g., sedentary behavior, environmental triggers) must be scaled. By bridging gaps in prevention and equitable care, policymakers can mitigate the lifelong disability and societal costs of migraine, safeguarding quality of life for vulnerable populations.

## Data Availability

The original contributions presented in the study are included in the article/Supplementary material, further inquiries can be directed to the corresponding author.

## Footnotes

^1^ https://vizhub.healthdata.org/gbd-results/
