# Supplementary material for: Rising global burden of migraine among adolescents and young adults: a 30-year analysis (1990–2021)
Source: Front Neurol. 2025 Sep 1;16:1652468. doi: 10.3389/fneur.2025.1652468 (PMC12434965; doi:10.3389/fneur.2025.1652468)
Supplement: Supplementary file 1 [file Image_1.pdf]

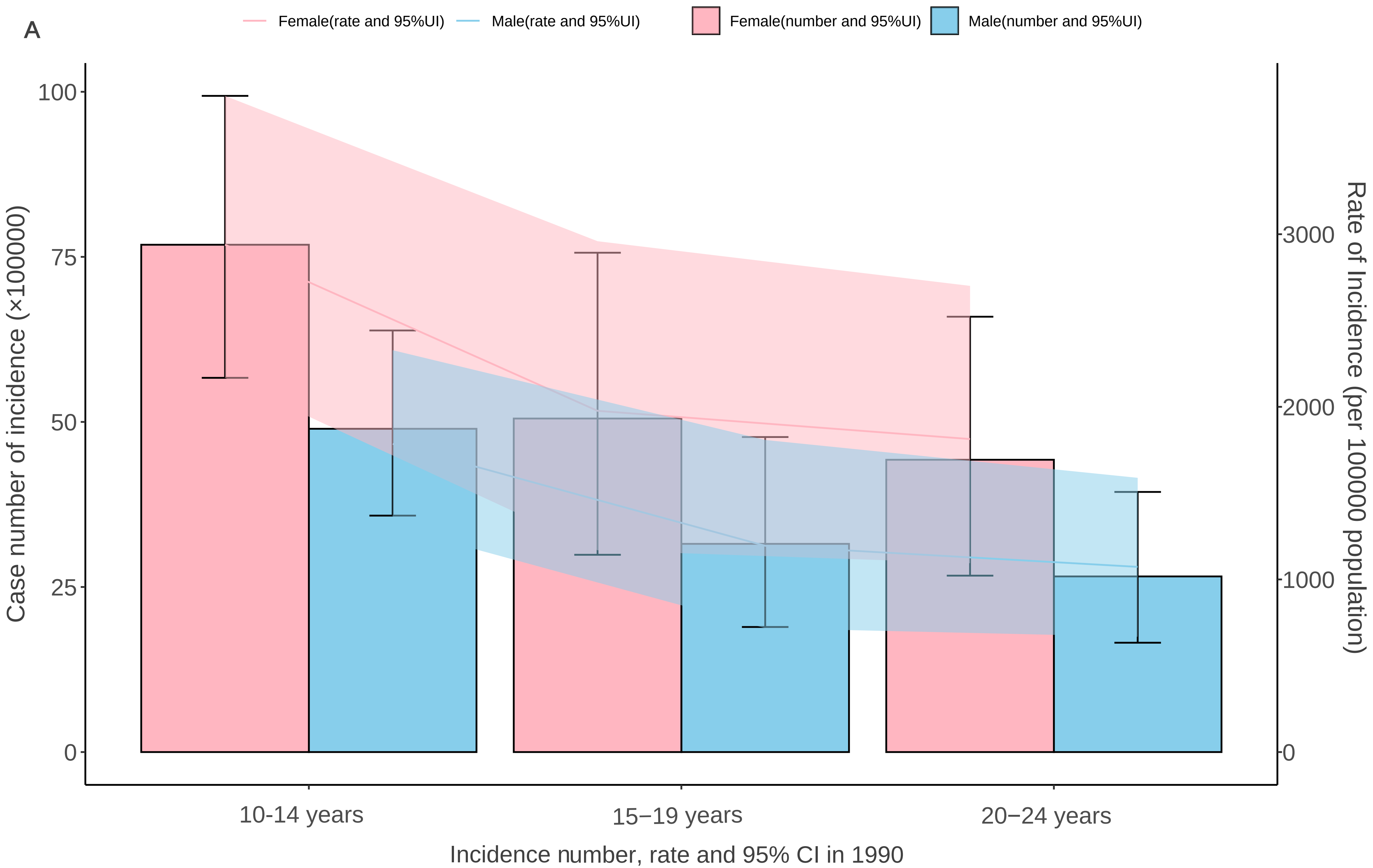
A
Female(rate and 95%UI)
Male(rate and 95%UI)
Female(number and 95%UI)
Male(number and 95%UI)
Case number of incidence (×100000)
100
75
50
25
0
Rate of Incidence (per 100000 population)
3000
2000
1000
0
10-14 years
15−19 years
20−24 years
Incidence number, rate and 95% CI in 1990

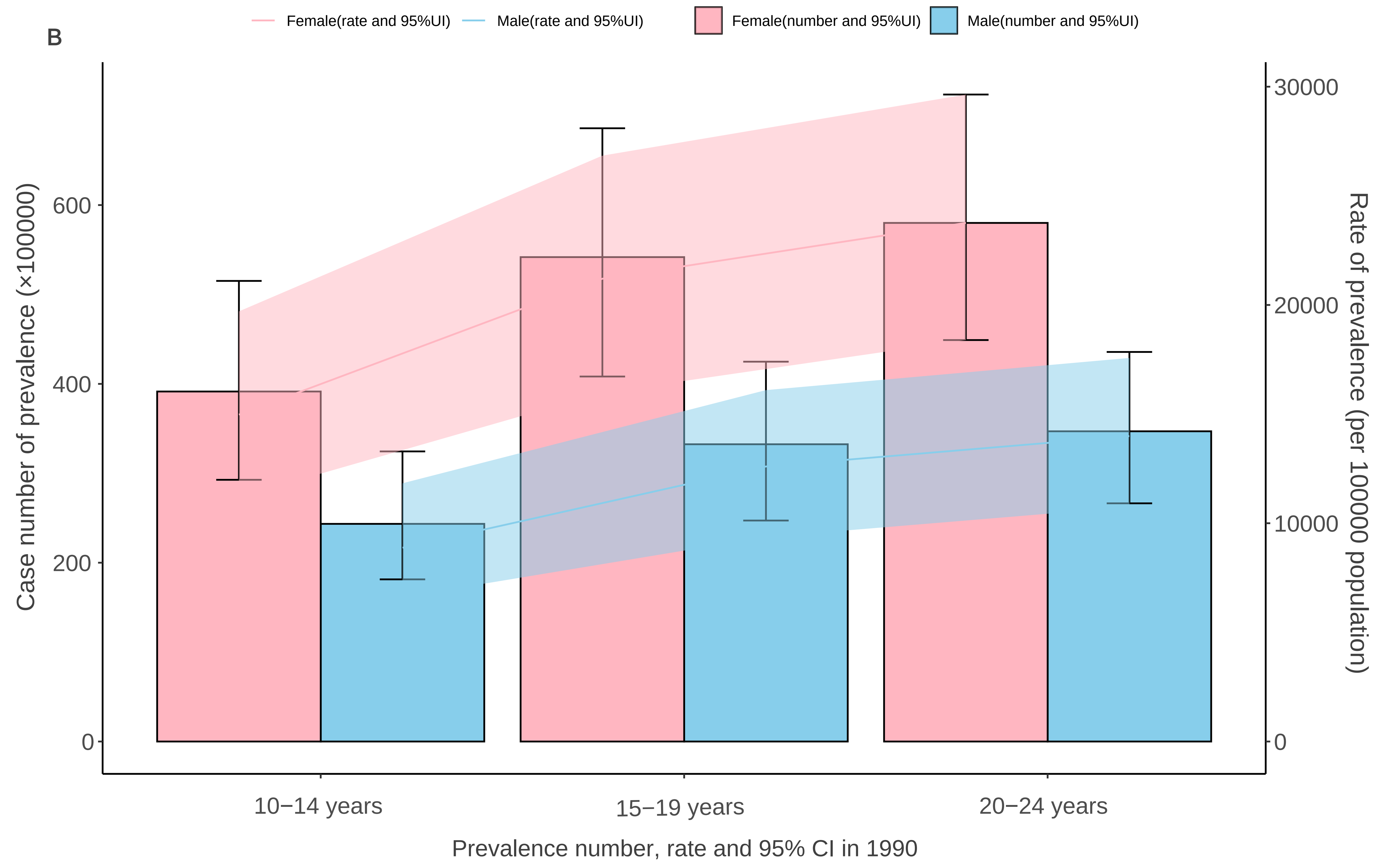
Female(rate and 95%UI)
Male(rate and 95%UI)
Female(number and 95%UI)
Male(number and 95%UI)
B
Case number of prevalence (×100000)
Rate of prevalence (per 100000 population)
0
200
400
600
0
10000
20000
30000
10−14 years
15−19 years
20−24 years
Prevalence number, rate and 95% CI in 1990

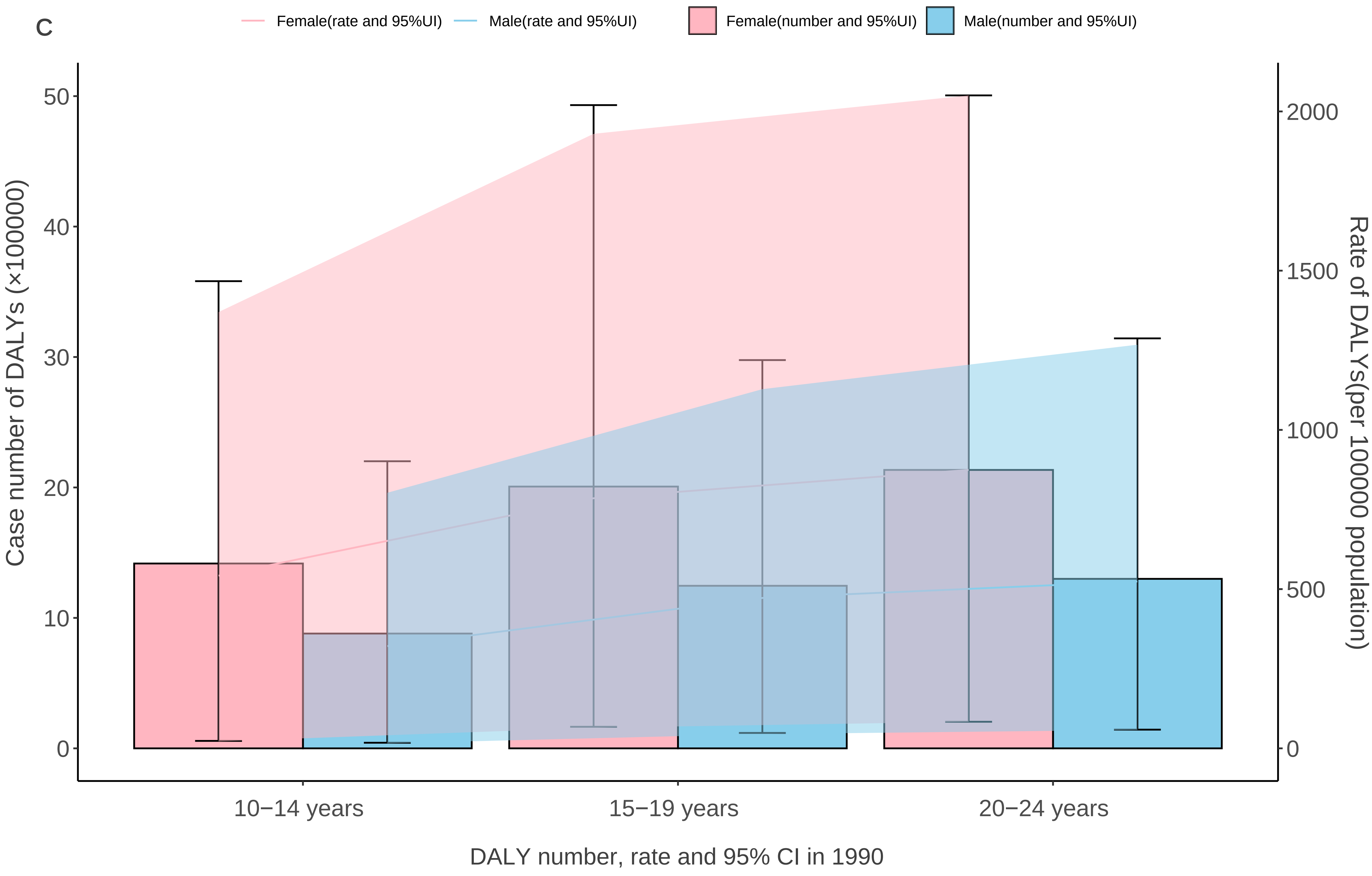
C
Female(rate and 95%UI)
Male(rate and 95%UI)
Female(number and 95%UI)
Male(number and 95%UI)
Case number of DALYs (×100000)
Rate of DALYs(per 100000 population)
0
10
20
30
40
50
500
1000
1500
2000
10−14 years
15−19 years
20−24 years
DALY number, rate and 95% CI in 1990

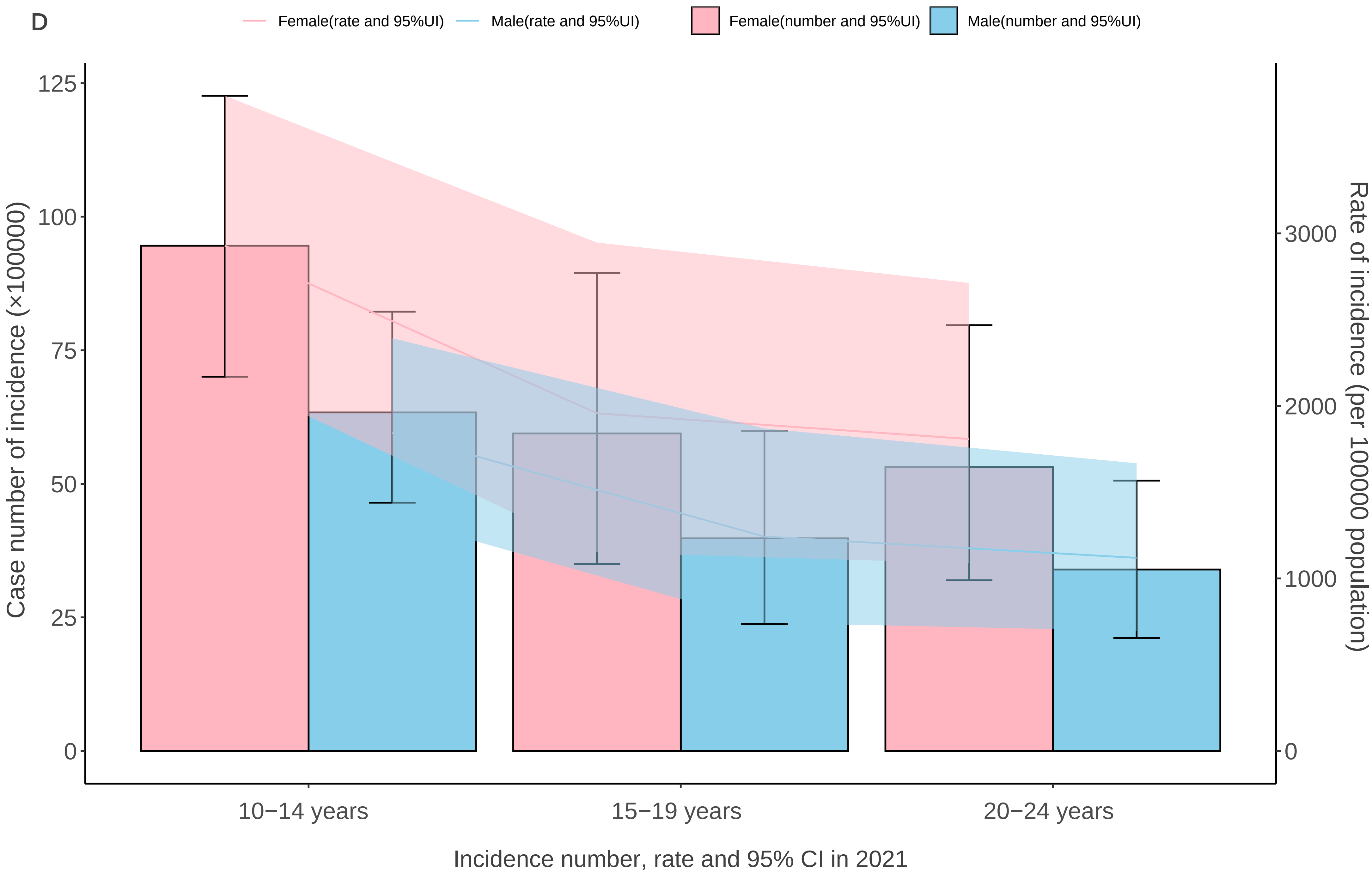
D
Female(rate and 95%UI)
Male(rate and 95%UI)
Female(number and 95%UI)
Male(number and 95%UI)
Case number of incidence (×100000)
Rate of incidence (per 100000 population)
125
100
75
50
25
0
3000
2000
1000
0
10−14 years
15−19 years
20−24 years
Incidence number, rate and 95% CI in 2021

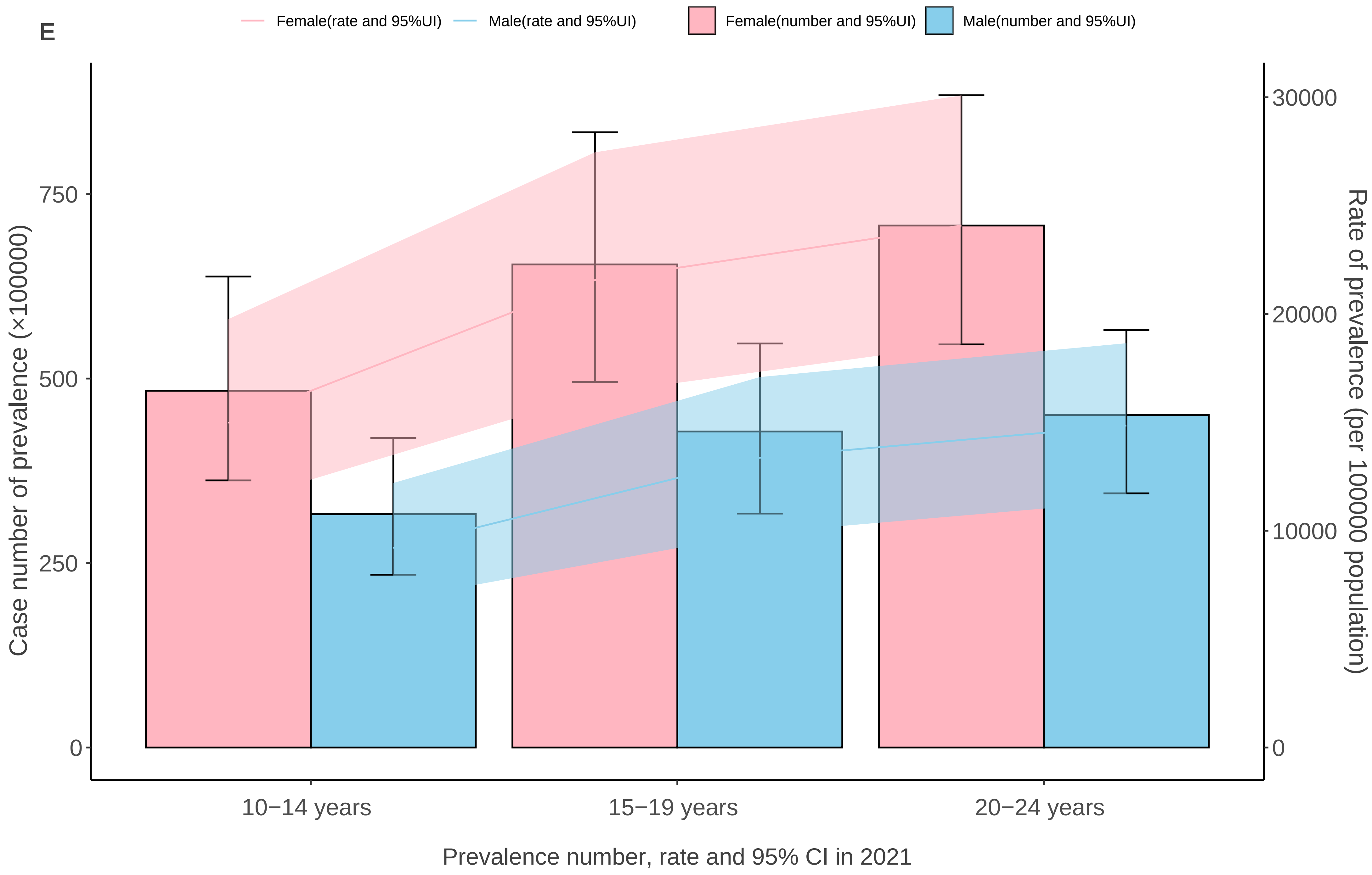

E
Female(rate and 95%UI)
Male(rate and 95%UI)
Female(number and 95%UI)
Male(number and 95%UI)
Case number of prevalence (×100000)
0
250
500
750
Rate of prevalence (per 100000 population)
0
10000
20000
30000
10−14 years
15−19 years
20−24 years
Prevalence number, rate and 95% CI in 2021

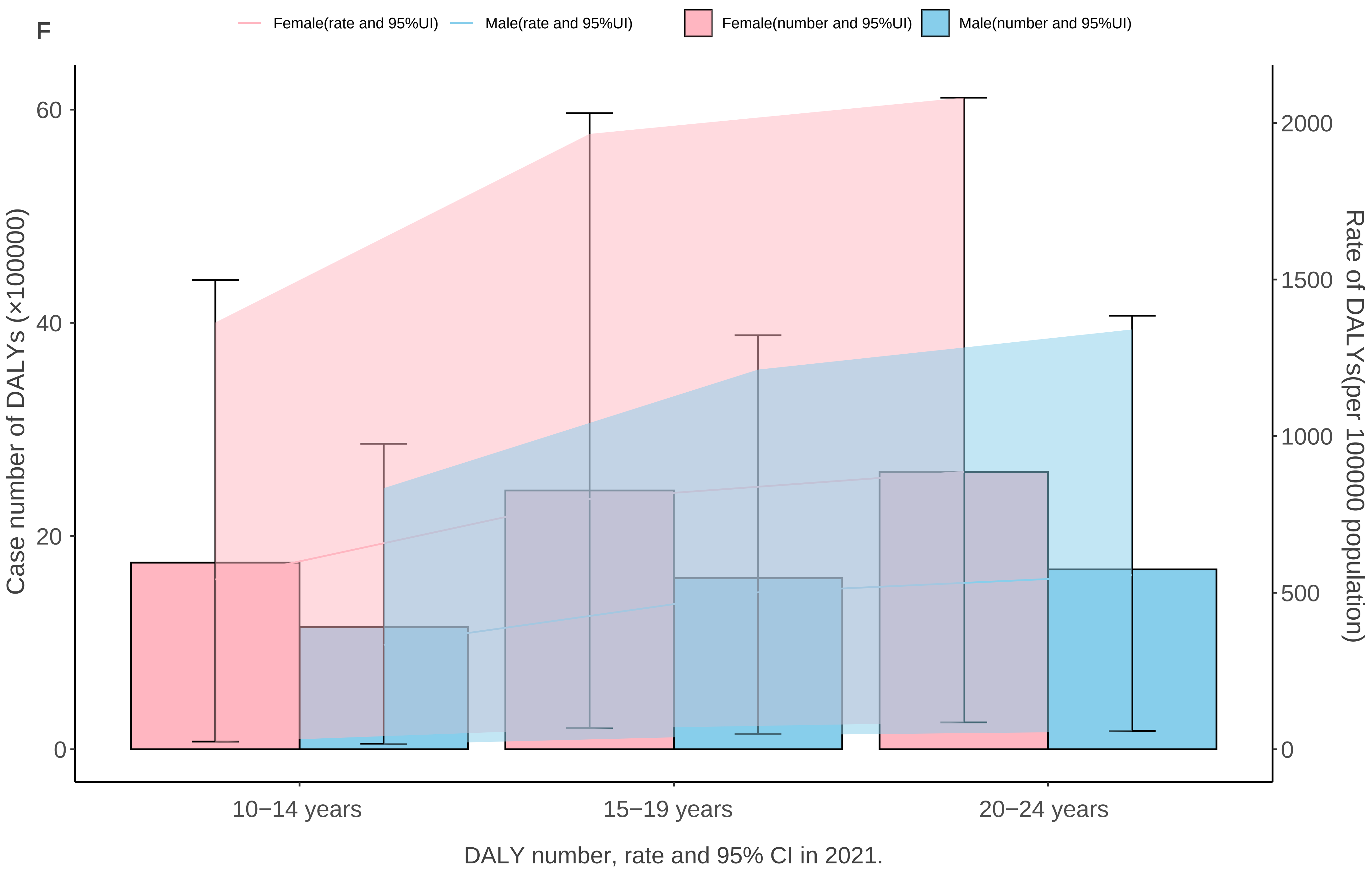

Supplementary figure 1: Global incidence, prevalence, DALY numbers, rates and 95% CI of migraine for male and female in 1990 and 2021: (A) Incidence number, rate and 95% CI in 1990; (B) Prevalence number, rate and 95% CI in 1990; (C) DALY number, rate and 95% CI in 1990; (D) Incidence number, rate and 95% CI in 2021; (E) Prevalence number, rate and 95% CI in 2021; (F) DALY number, rate and 95% CI in 2021.
