# Supplementary material for: Rising global burden of migraine among adolescents and young adults: a 30-year analysis (1990–2021)
Source: Front Neurol. 2025 Sep 1;16:1652468. doi: 10.3389/fneur.2025.1652468 (PMC12434965; doi:10.3389/fneur.2025.1652468)
Supplement: Supplementary file 2 [file Image_2.pdf]

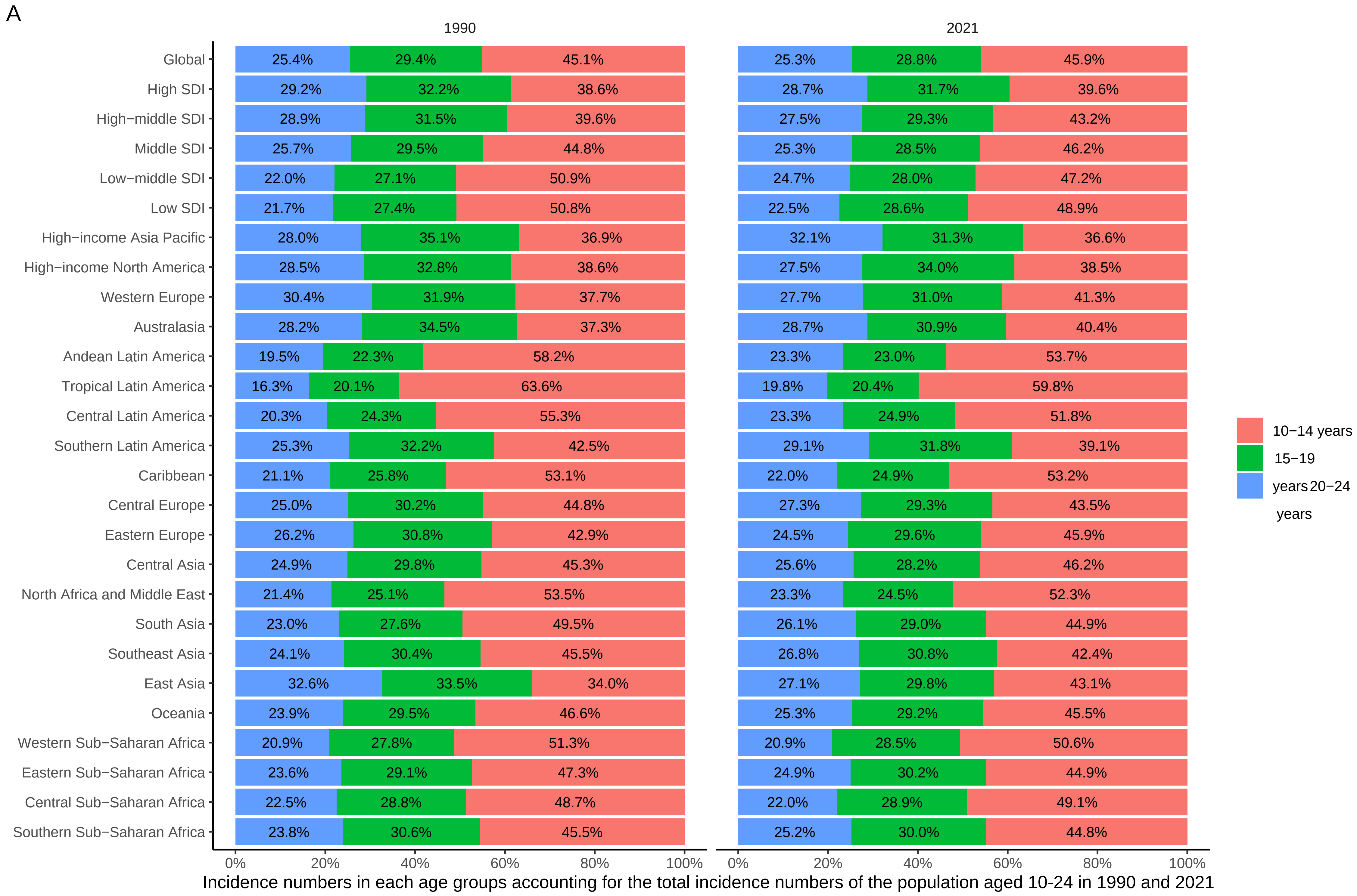
A
1990
2021
Global
High SDI
High−middle SDI
Middle SDI
Low−middle SDI
Low SDI
High−income Asia Pacific
High−income North America
Western Europe
Australasia
Andean Latin America
Tropical Latin America
Central Latin America
Southern Latin America
Caribbean
Central Europe
Eastern Europe
Central Asia
North Africa and Middle East
South Asia
Southeast Asia
East Asia
Oceania
Western Sub−Saharan Africa
Eastern Sub−Saharan Africa
Central Sub−Saharan Africa
Southern Sub−Saharan Africa
25.4% 29.4% 45.1% | 25.3% 28.8% 45.9%
29.2% 32.2% 38.6% | 28.7% 31.7% 39.6%
28.9% 31.5% 39.6% | 27.5% 29.3% 43.2%
25.7% 29.5% 44.8% | 25.3% 28.5% 46.2%
22.0% 27.1% 50.9% | 24.7% 28.0% 47.2%
21.7% 27.4% 50.8% | 22.5% 28.6% 48.9%
28.0% 35.1% 36.9% | 32.1% 31.3% 36.6%
28.5% 32.8% 38.6% | 27.5% 34.0% 38.5%
30.4% 31.9% 37.7% | 27.7% 31.0% 41.3%
28.2% 34.5% 37.3% | 28.7% 30.9% 40.4%
19.5% 22.3% 58.2% | 23.3% 23.0% 53.7%
16.3% 20.1% 63.6% | 19.8% 20.4% 59.8%
20.3% 24.3% 55.3% | 23.3% 24.9% 51.8%
25.3% 32.2% 42.5% | 29.1% 31.8% 39.1%
21.1% 25.8% 53.1% | 22.0% 24.9% 53.2%
25.0% 30.2% 44.8% | 27.3% 29.3% 43.5%
26.2% 30.8% 42.9% | 24.5% 29.6% 45.9%
24.9% 29.8% 45.3% | 25.6% 28.2% 46.2%
21.4% 25.1% 53.5% | 23.3% 24.5% 52.3%
23.0% 27.6% 49.5% | 26.1% 29.0% 44.9%
24.1% 30.4% 45.5% | 26.8% 30.8% 42.4%
32.6% 33.5% 34.0% | 27.1% 29.8% 43.1%
23.9% 29.5% 46.6% | 25.3% 29.2% 45.5%
20.9% 27.8% 51.3% | 20.9% 28.5% 50.6%
23.6% 29.1% 47.3% | 24.9% 30.2% 44.9%
22.5% 28.8% 48.7% | 22.0% 28.9% 49.1%
23.8% 30.6% 45.5% | 25.2% 30.0% 44.8%
0% 20% 40% 60% 80% 100%
10−14 years
15−19 years
20−24 years
Incidence numbers in each age groups accounting for the total incidence numbers of the population aged 10-24 in 1990 and 2021

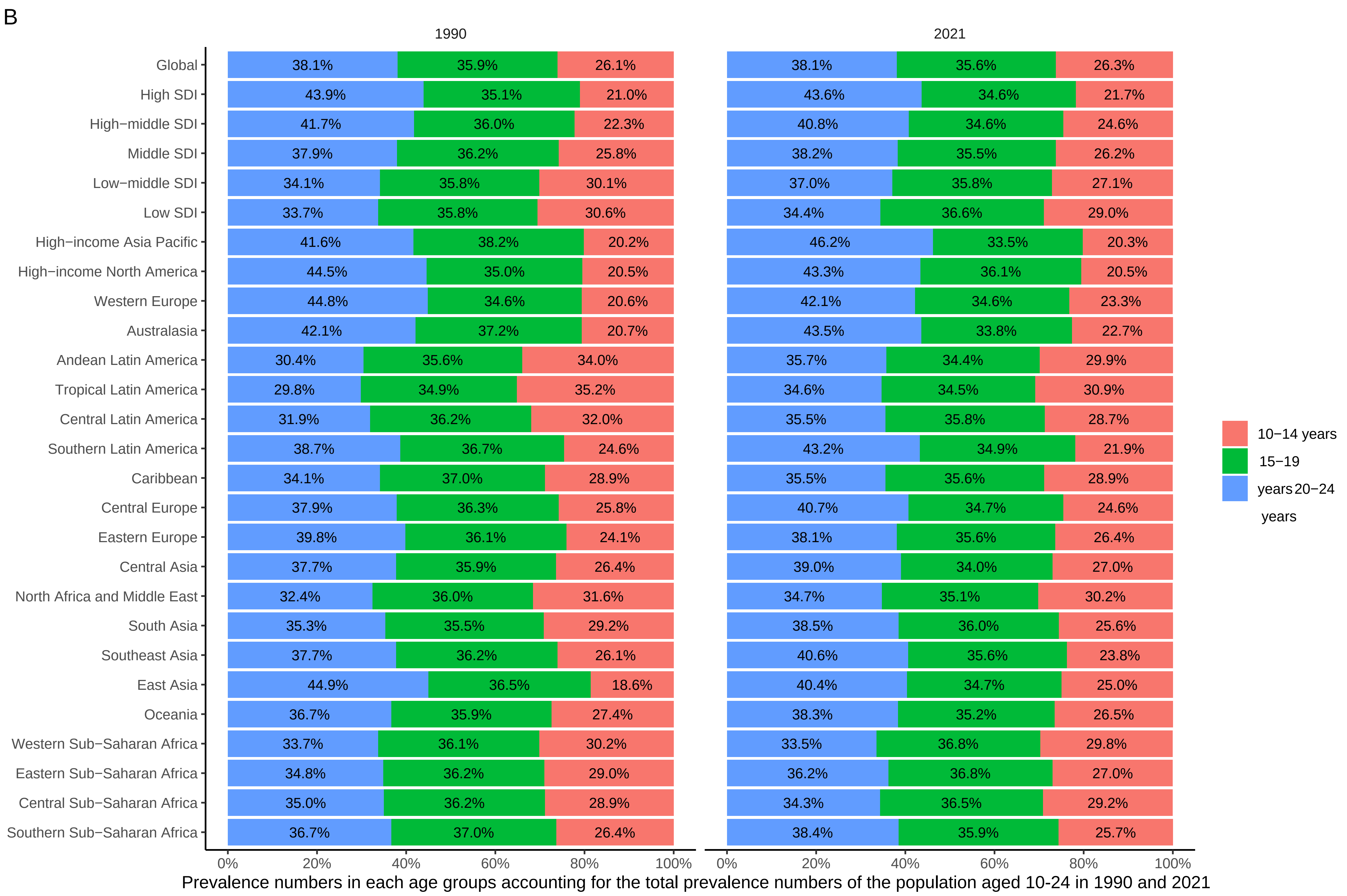
B
1990
2021
Global
High SDI
High−middle SDI
Middle SDI
Low−middle SDI
Low SDI
High−income Asia Pacific
High−income North America
Western Europe
Australasia
Andean Latin America
Tropical Latin America
Central Latin America
Southern Latin America
Caribbean
Central Europe
Eastern Europe
Central Asia
North Africa and Middle East
South Asia
Southeast Asia
East Asia
Oceania
Western Sub−Saharan Africa
Eastern Sub−Saharan Africa
Central Sub−Saharan Africa
Southern Sub−Saharan Africa
38.1% 35.9% 26.1% | 38.1% 35.6% 26.3%
43.9% 35.1% 21.0% | 43.6% 34.6% 21.7%
41.7% 36.0% 22.3% | 40.8% 34.6% 24.6%
37.9% 36.2% 25.8% | 38.2% 35.5% 26.2%
34.1% 35.8% 30.1% | 37.0% 35.8% 27.1%
33.7% 35.8% 30.6% | 34.4% 36.6% 29.0%
41.6% 38.2% 20.2% | 46.2% 33.5% 20.3%
44.5% 35.0% 20.5% | 43.3% 36.1% 20.5%
44.8% 34.6% 20.6% | 42.1% 34.6% 23.3%
42.1% 37.2% 20.7% | 43.5% 33.8% 22.7%
30.4% 35.6% 34.0% | 35.7% 34.4% 29.9%
29.8% 34.9% 35.2% | 34.6% 34.5% 30.9%
31.9% 36.2% 32.0% | 35.5% 35.8% 28.7%
38.7% 36.7% 24.6% | 43.2% 34.9% 21.9%
34.1% 37.0% 28.9% | 35.5% 35.6% 28.9%
37.9% 36.3% 25.8% | 40.7% 34.7% 24.6%
39.8% 36.1% 24.1% | 38.1% 35.6% 26.4%
37.7% 35.9% 26.4% | 39.0% 34.0% 27.0%
32.4% 36.0% 31.6% | 34.7% 35.1% 30.2%
35.3% 35.5% 29.2% | 38.5% 36.0% 25.6%
37.7% 36.2% 26.1% | 40.6% 35.6% 23.8%
44.9% 36.5% 18.6% | 40.4% 34.7% 25.0%
36.7% 35.9% 27.4% | 38.3% 35.2% 26.5%
33.7% 36.1% 30.2% | 33.5% 36.8% 29.8%
34.8% 36.2% 29.0% | 36.2% 36.8% 27.0%
35.0% 36.2% 28.9% | 34.3% 36.5% 29.2%
36.7% 37.0% 26.4% | 38.4% 35.9% 25.7%
0% 20% 40% 60% 80% 100%
10−14 years
15−19
years 20−24
years
Prevalence numbers in each age groups accounting for the total prevalence numbers of the population aged 10-24 in 1990 and 2021

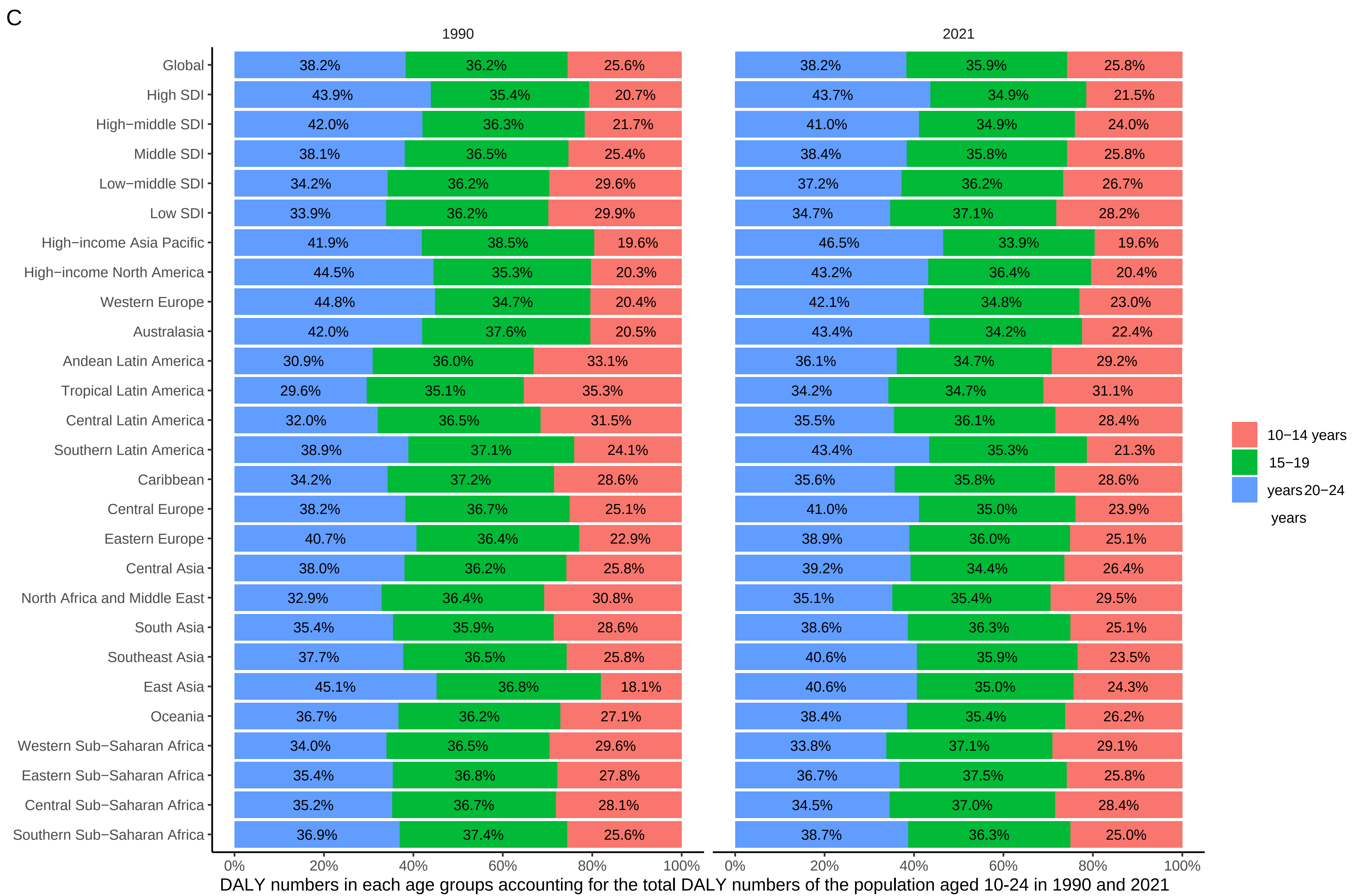
C
1990
2021
Global
High SDI
High−middle SDI
Middle SDI
Low−middle SDI
Low SDI
High−income Asia Pacific
High−income North America
Western Europe
Australasia
Andean Latin America
Tropical Latin America
Central Latin America
Southern Latin America
Caribbean
Central Europe
Eastern Europe
Central Asia
North Africa and Middle East
South Asia
Southeast Asia
East Asia
Oceania
Western Sub−Saharan Africa
Eastern Sub−Saharan Africa
Central Sub−Saharan Africa
Southern Sub−Saharan Africa
10−14 years
15−19 years
20−24 years
0%
20%
40%
60%
80%
100%
DALY numbers in each age groups accounting for the total DALY numbers of the population aged 10-24 in 1990 and 2021

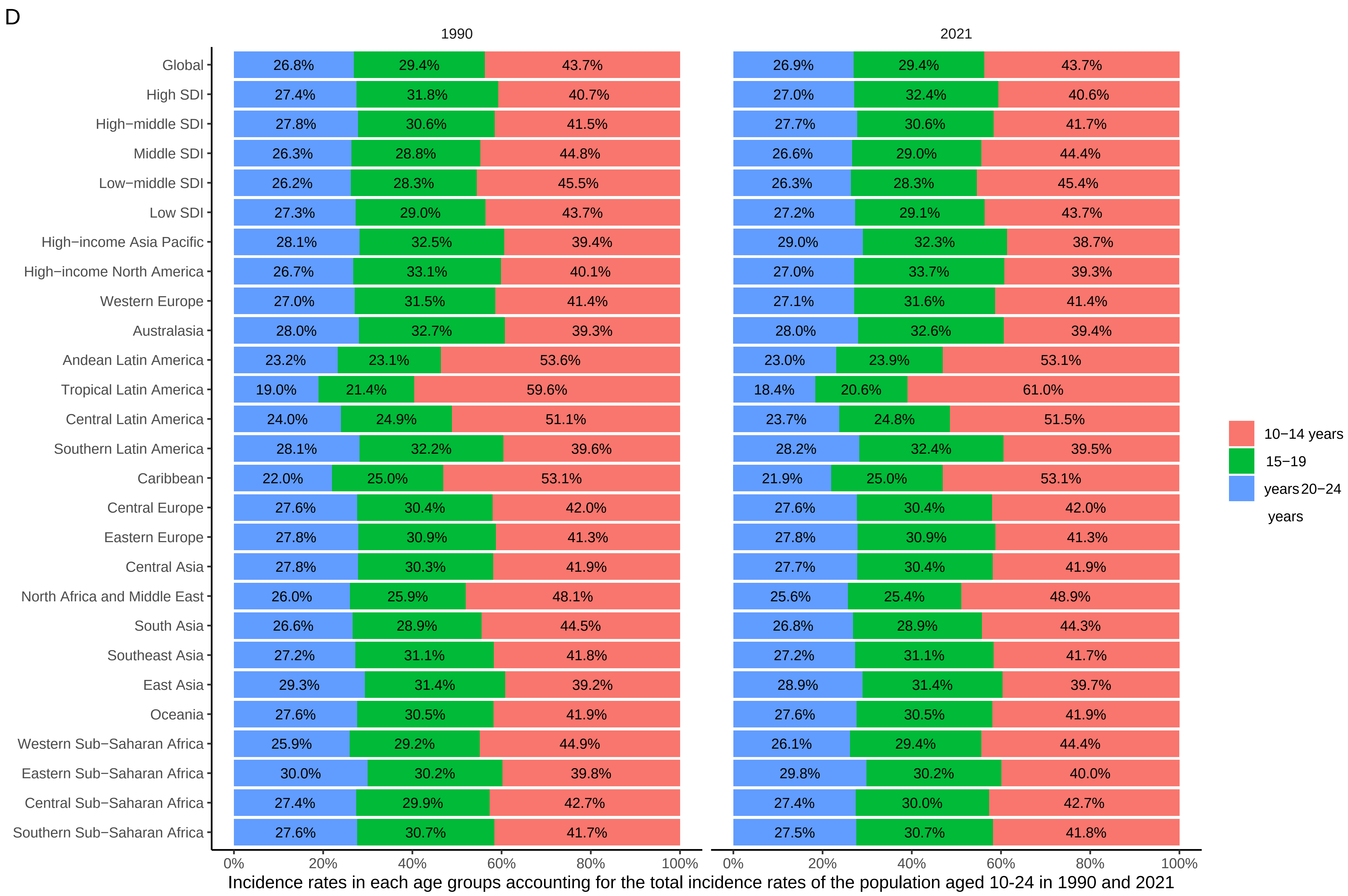
D
1990
2021
Global 26.8% 29.4% 43.7% | 26.9% 29.4% 43.7%
High SDI 27.4% 31.8% 40.7% | 27.0% 32.4% 40.6%
High−middle SDI 27.8% 30.6% 41.5% | 27.7% 30.6% 41.7%
Middle SDI 26.3% 28.8% 44.8% | 26.6% 29.0% 44.4%
Low−middle SDI 26.2% 28.3% 45.5% | 26.3% 28.3% 45.4%
Low SDI 27.3% 29.0% 43.7% | 27.2% 29.1% 43.7%
High−income Asia Pacific 28.1% 32.5% 39.4% | 29.0% 32.3% 38.7%
High−income North America 26.7% 33.1% 40.1% | 27.0% 33.7% 39.3%
Western Europe 27.0% 31.5% 41.4% | 27.1% 31.6% 41.4%
Australasia 28.0% 32.7% 39.3% | 28.0% 32.6% 39.4%
Andean Latin America 23.2% 23.1% 53.6% | 23.0% 23.9% 53.1%
Tropical Latin America 19.0% 21.4% 59.6% | 18.4% 20.6% 61.0%
Central Latin America 24.0% 24.9% 51.1% | 23.7% 24.8% 51.5%
Southern Latin America 28.1% 32.2% 39.6% | 28.2% 32.4% 39.5%
Caribbean 22.0% 25.0% 53.1% | 21.9% 25.0% 53.1%
Central Europe 27.6% 30.4% 42.0% | 27.6% 30.4% 42.0%
Eastern Europe 27.8% 30.9% 41.3% | 27.8% 30.9% 41.3%
Central Asia 27.8% 30.3% 41.9% | 27.7% 30.4% 41.9%
North Africa and Middle East 26.0% 25.9% 48.1% | 25.6% 25.4% 48.9%
South Asia 26.6% 28.9% 44.5% | 26.8% 28.9% 44.3%
Southeast Asia 27.2% 31.1% 41.8% | 27.2% 31.1% 41.7%
East Asia 29.3% 31.4% 39.2% | 28.9% 31.4% 39.7%
Oceania 27.6% 30.5% 41.9% | 27.6% 30.5% 41.9%
Western Sub−Saharan Africa 25.9% 29.2% 44.9% | 26.1% 29.4% 44.4%
Eastern Sub−Saharan Africa 30.0% 30.2% 39.8% | 29.8% 30.2% 40.0%
Central Sub−Saharan Africa 27.4% 29.9% 42.7% | 27.4% 30.0% 42.7%
Southern Sub−Saharan Africa 27.6% 30.7% 41.7% | 27.5% 30.7% 41.8%
0% 20% 40% 60% 80% 100%
10−14 years
15−19 years
20−24 years
Incidence rates in each age groups accounting for the total incidence rates of the population aged 10-24 in 1990 and 2021

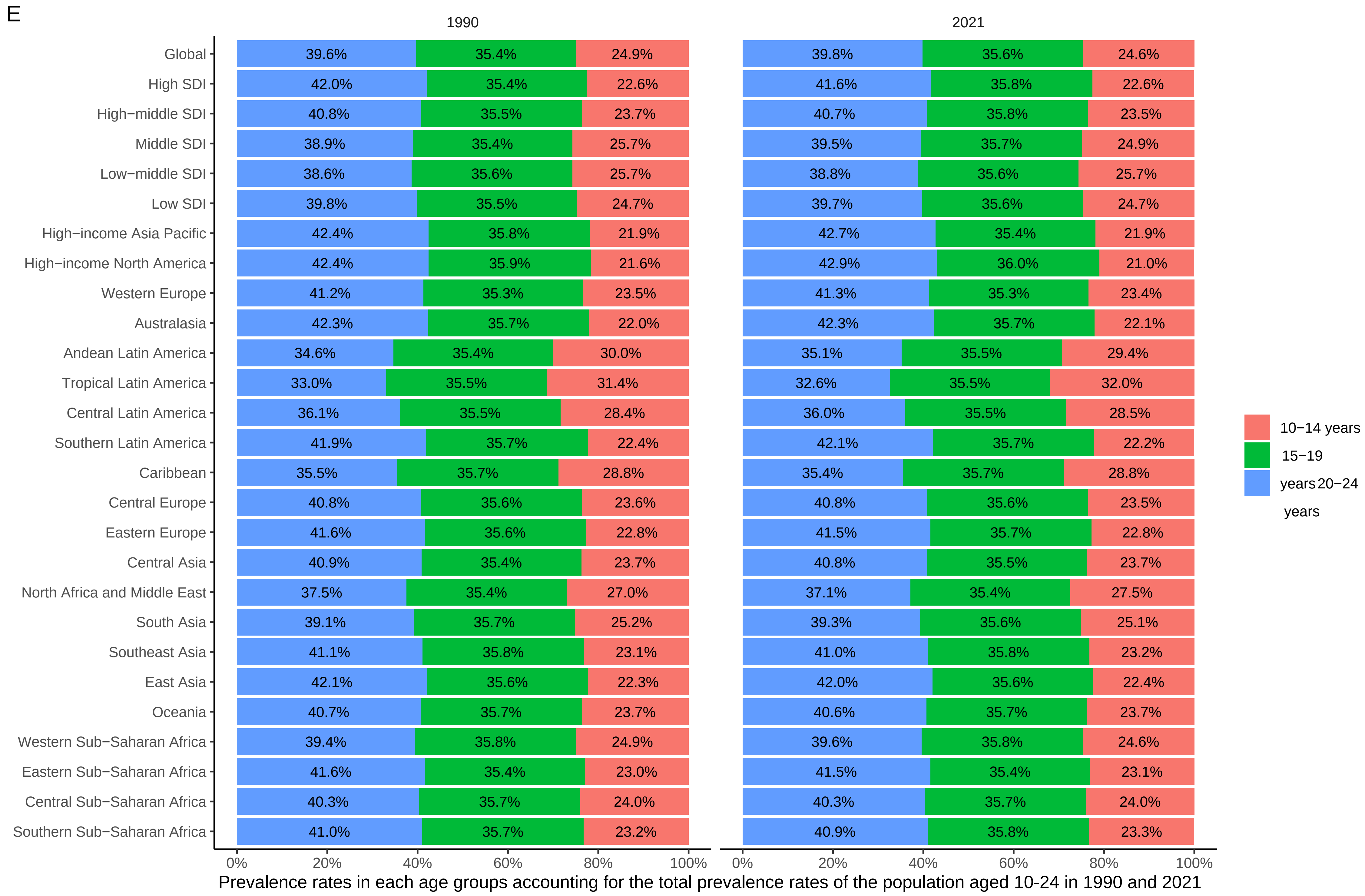
E
1990
2021
Global
High SDI
High−middle SDI
Middle SDI
Low−middle SDI
Low SDI
High−income Asia Pacific
High−income North America
Western Europe
Australasia
Andean Latin America
Tropical Latin America
Central Latin America
Southern Latin America
Caribbean
Central Europe
Eastern Europe
Central Asia
North Africa and Middle East
South Asia
Southeast Asia
East Asia
Oceania
Western Sub−Saharan Africa
Eastern Sub−Saharan Africa
Central Sub−Saharan Africa
Southern Sub−Saharan Africa
39.6% 35.4% 24.9% 39.8% 35.6% 24.6%
42.0% 35.4% 22.6% 41.6% 35.8% 22.6%
40.8% 35.5% 23.7% 40.7% 35.8% 23.5%
38.9% 35.4% 25.7% 39.5% 35.7% 24.9%
38.6% 35.6% 25.7% 38.8% 35.6% 25.7%
39.8% 35.5% 24.7% 39.7% 35.6% 24.7%
42.4% 35.8% 21.9% 42.7% 35.4% 21.9%
42.4% 35.9% 21.6% 42.9% 36.0% 21.0%
41.2% 35.3% 23.5% 41.3% 35.3% 23.4%
42.3% 35.7% 22.0% 42.3% 35.7% 22.1%
34.6% 35.4% 30.0% 35.1% 35.5% 29.4%
33.0% 35.5% 31.4% 32.6% 35.5% 32.0%
36.1% 35.5% 28.4% 36.0% 35.5% 28.5%
41.9% 35.7% 22.4% 42.1% 35.7% 22.2%
35.5% 35.7% 28.8% 35.4% 35.7% 28.8%
40.8% 35.6% 23.6% 40.8% 35.6% 23.5%
41.6% 35.6% 22.8% 41.5% 35.7% 22.8%
40.9% 35.4% 23.7% 40.8% 35.5% 23.7%
37.5% 35.4% 27.0% 37.1% 35.4% 27.5%
39.1% 35.7% 25.2% 39.3% 35.6% 25.1%
41.1% 35.8% 23.1% 41.0% 35.8% 23.2%
42.1% 35.6% 22.3% 42.0% 35.6% 22.4%
40.7% 35.7% 23.7% 40.6% 35.7% 23.7%
39.4% 35.8% 24.9% 39.6% 35.8% 24.6%
41.6% 35.4% 23.0% 41.5% 35.4% 23.1%
40.3% 35.7% 24.0% 40.3% 35.7% 24.0%
41.0% 35.7% 23.2% 40.9% 35.8% 23.3%
0% 20% 40% 60% 80% 100%
10−14 years
15−19
years 20−24
years
Prevalence rates in each age groups accounting for the total prevalence rates of the population aged 10-24 in 1990 and 2021

F

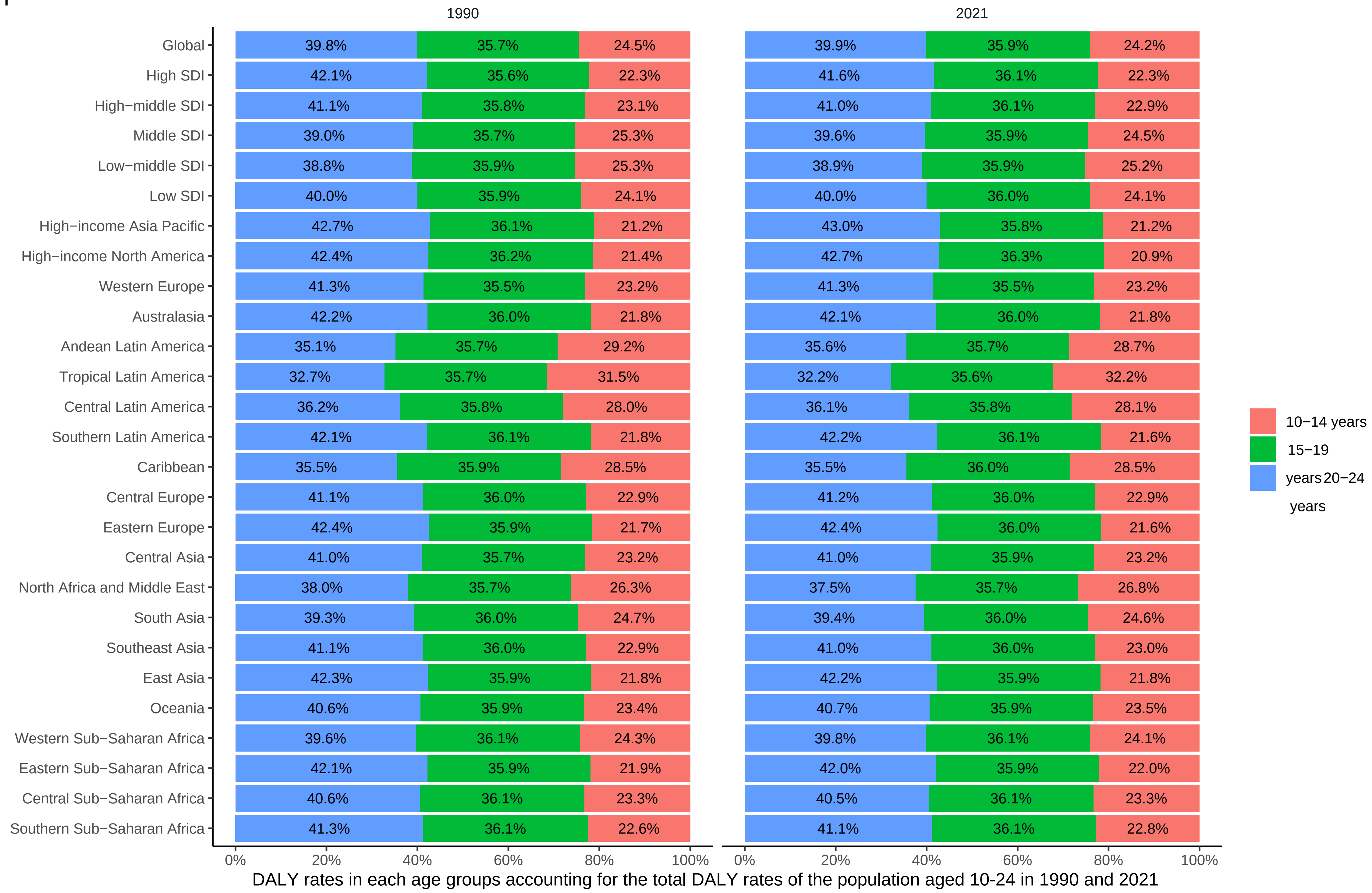

Supplementary figure 2: The percentage change of migraine incidence, prevalence, DALY numbers and rates in each age groups accounting for the total incidence, prevalence, DALY numbers and rates of the population aged 10-24 in 1990 and 2021, globally and by SDI quintile and GBD super-region: (A) incidence number; (B) prevalence number; (C) DALY number; (D) incidence rate; (E) prevalence rate; (F) DALY rate.
