# Supplementary material for: Rising global burden of migraine among adolescents and young adults: a 30-year analysis (1990–2021)
Source: Front Neurol. 2025 Sep 1;16:1652468. doi: 10.3389/fneur.2025.1652468 (PMC12434965; doi:10.3389/fneur.2025.1652468)
Supplement: Supplementary file 3 [file Image_3.pdf]

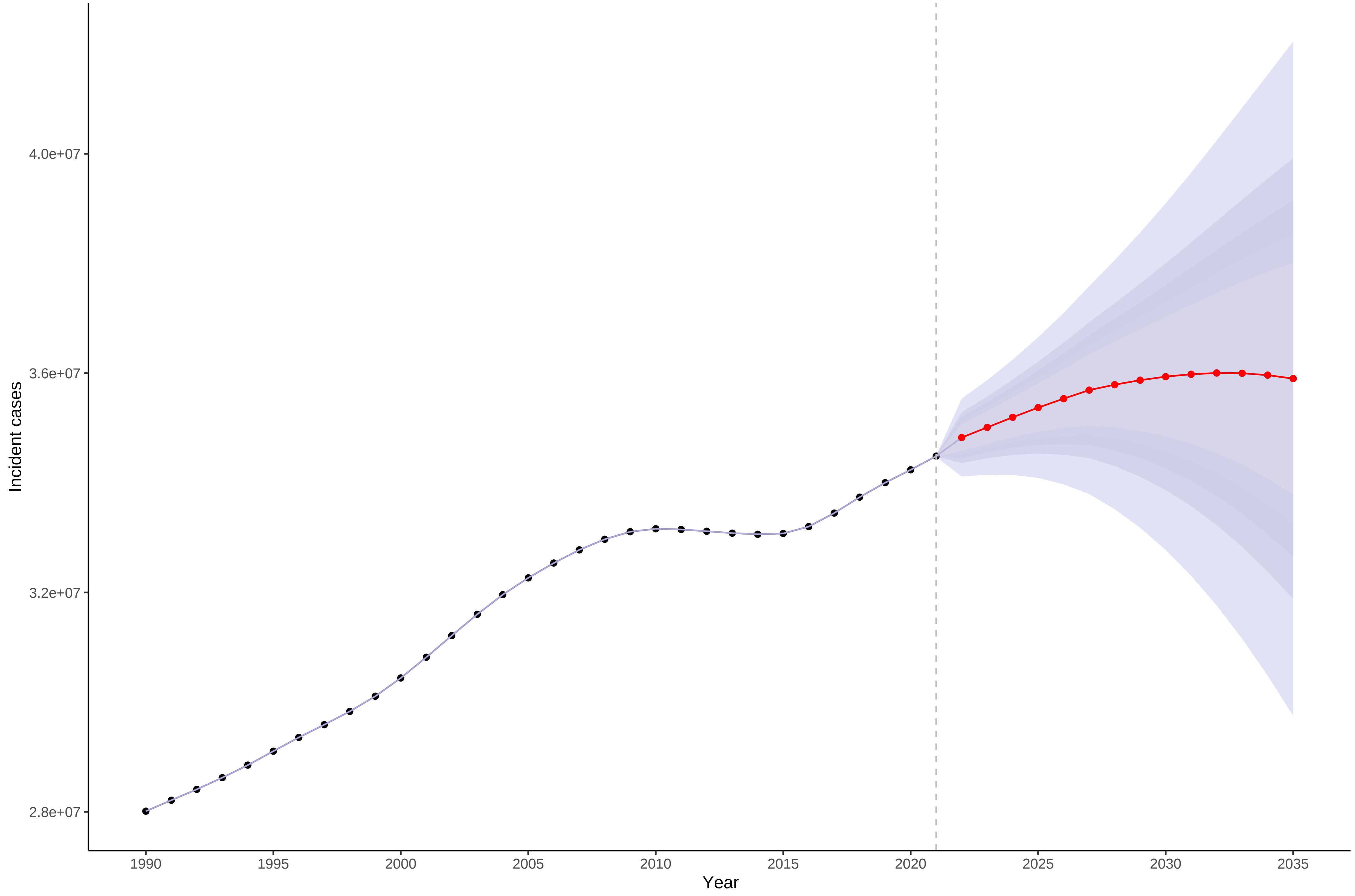

Incident cases
4.0e+07
3.6e+07
3.2e+07
2.8e+07
1990
1995
2000
2005
2010
2015
2020
2025
2030
2035
Year

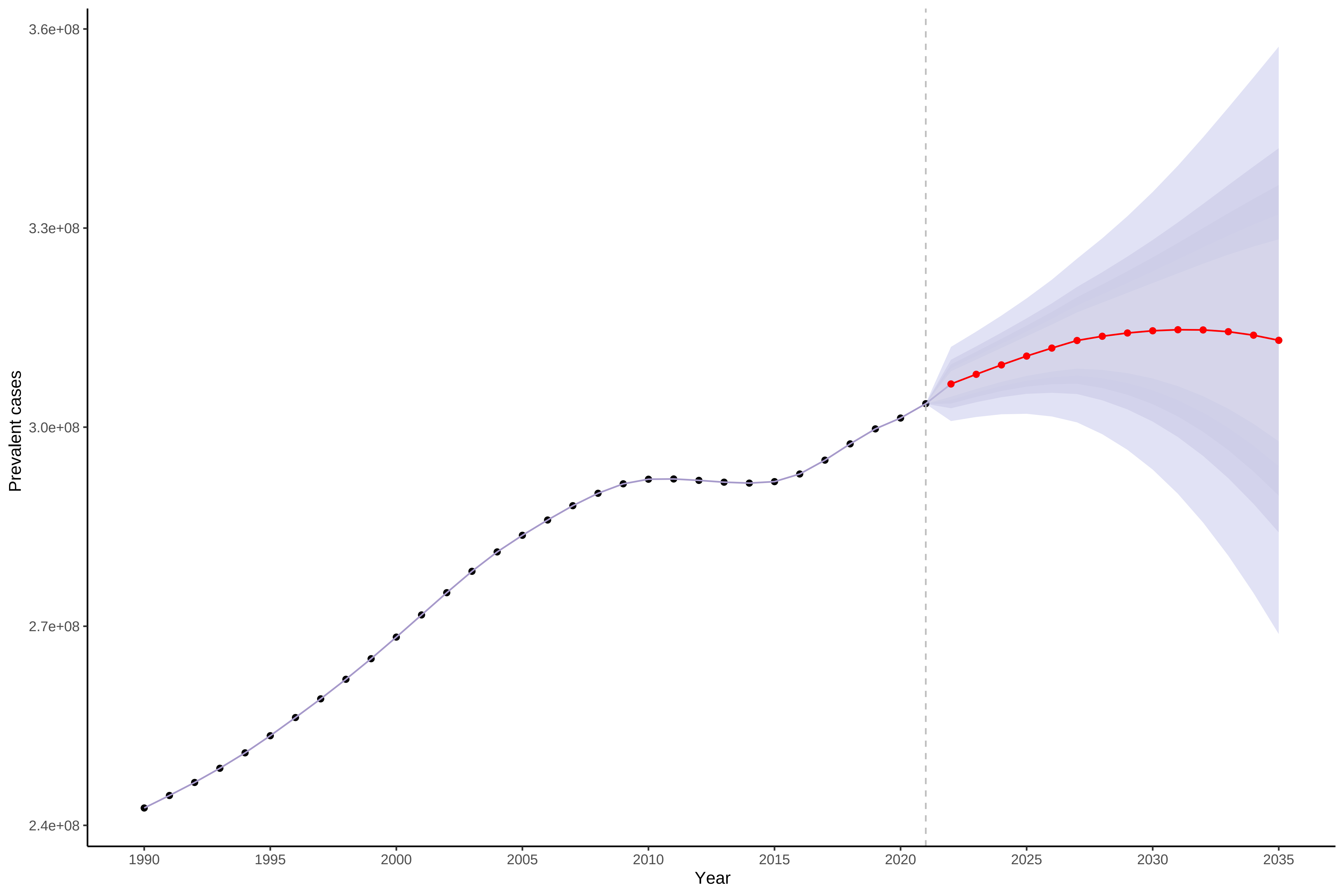

3.6e+08
3.3e+08
3.0e+08
2.7e+08
2.4e+08
Prevalent cases
1990
1995
2000
2005
2010
2015
2020
2025
2030
2035
Year

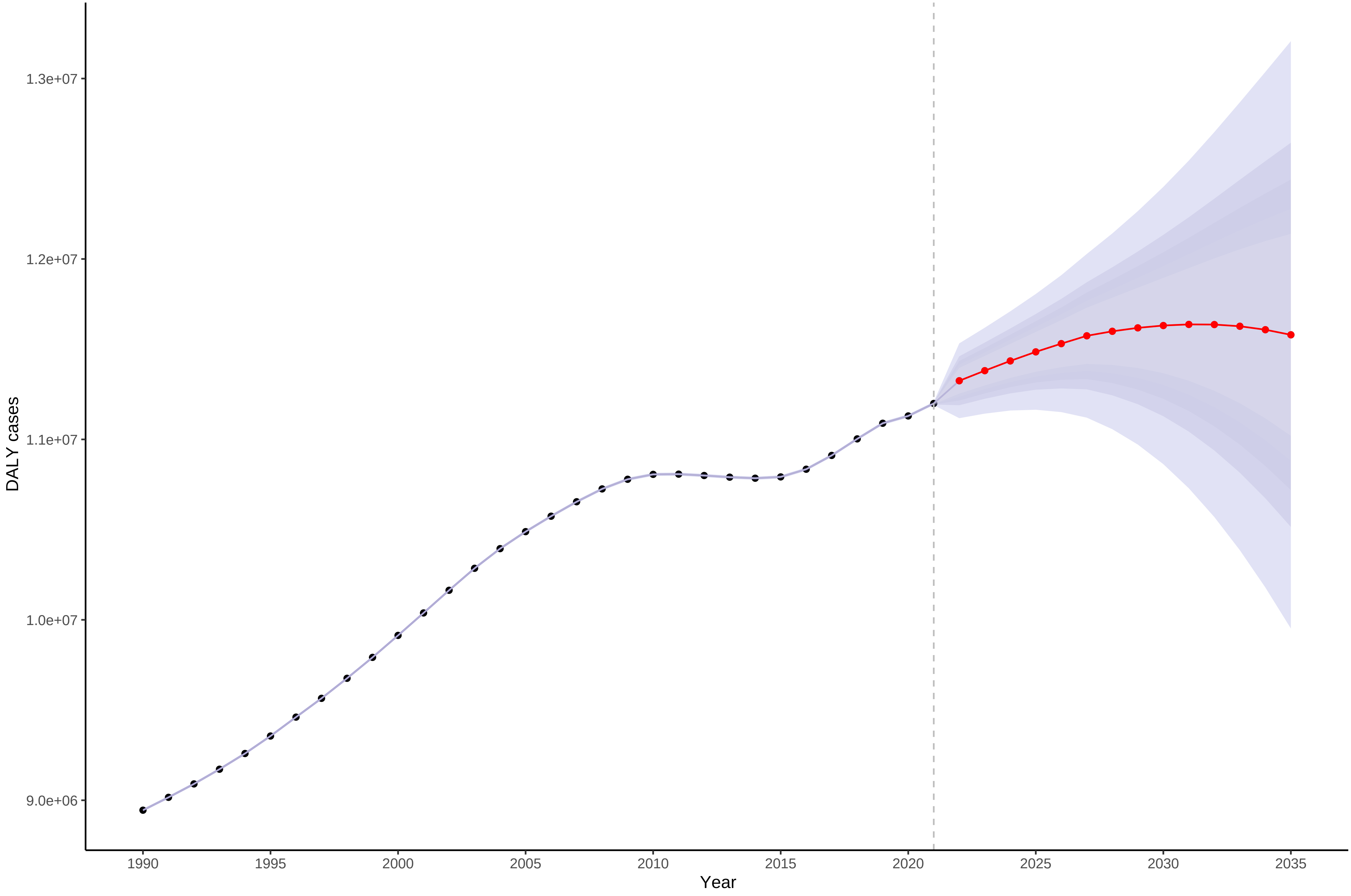

Supplementary figure 3: The global change trends of incident, prevalent, and DALY cases of migraine from 1990 to 2021, and its predicted tr ends between 2020 and 2035: (A) Incident cases; (B) Prevalent cases; (C) DALY cases.
