# Supplementary material for: Rising global burden of migraine among adolescents and young adults: a 30-year analysis (1990–2021)
Source: Front Neurol. 2025 Sep 1;16:1652468. doi: 10.3389/fneur.2025.1652468 (PMC12434965; doi:10.3389/fneur.2025.1652468)
Supplement: Supplementary file 4 [file Image_4.pdf]

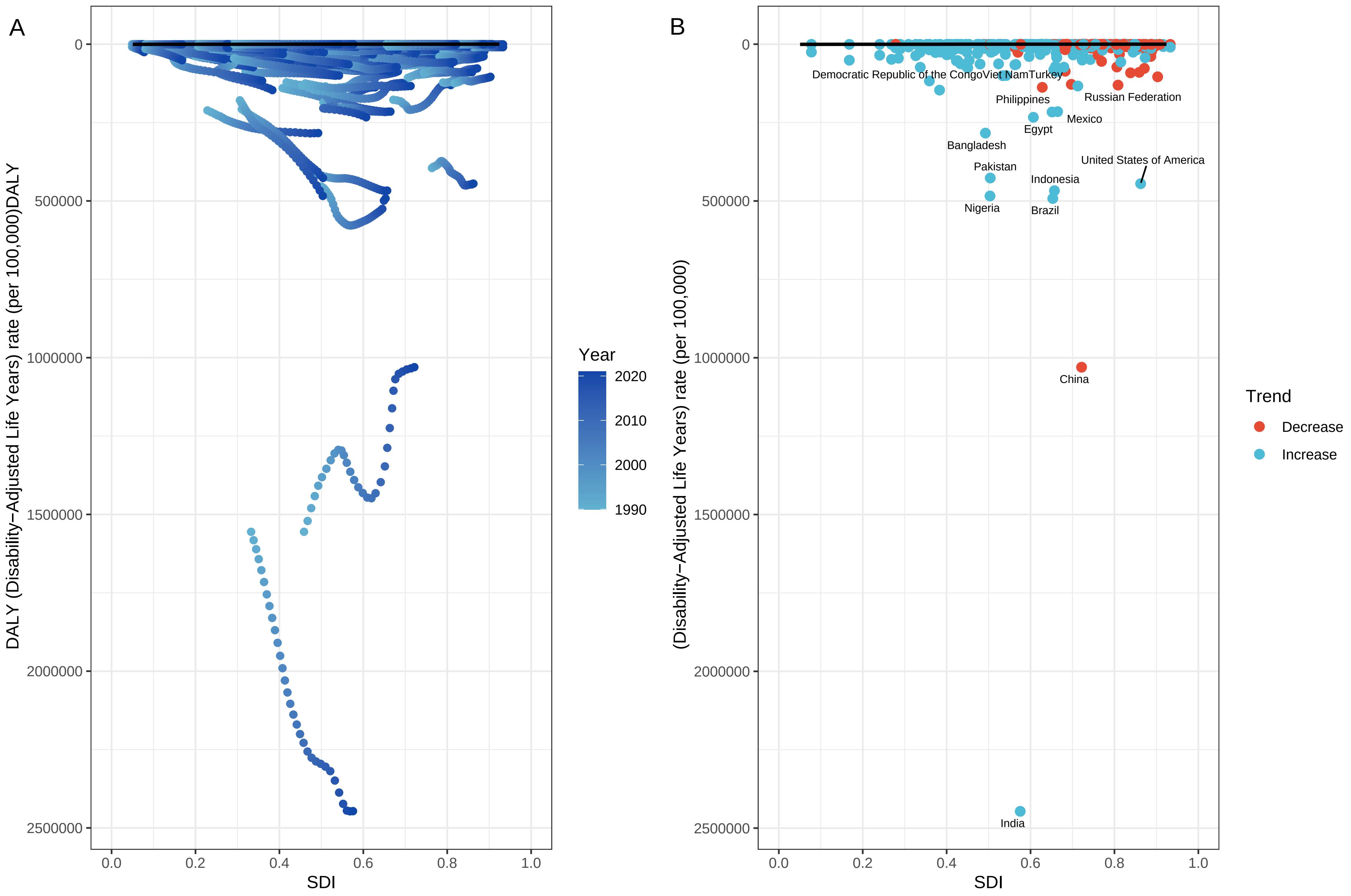

A
DALY (Disability−Adjusted Life Years) rate (per 100,000)DALY
0
500000
1000000
1500000
2000000
2500000
0.0
0.2
0.4
0.6
0.8
1.0
SDI
Year
2020
2010
2000
1990
B
(Disability−Adjusted Life Years) rate (per 100,000)
Democratic Republic of the CongoViet NamTurkey
Philippines
Russian Federation
Mexico
Egypt
Bangladesh
Pakistan
United States of America
Indonesia
Nigeria
Brazil
China
India
Trend
Decrease
Increase

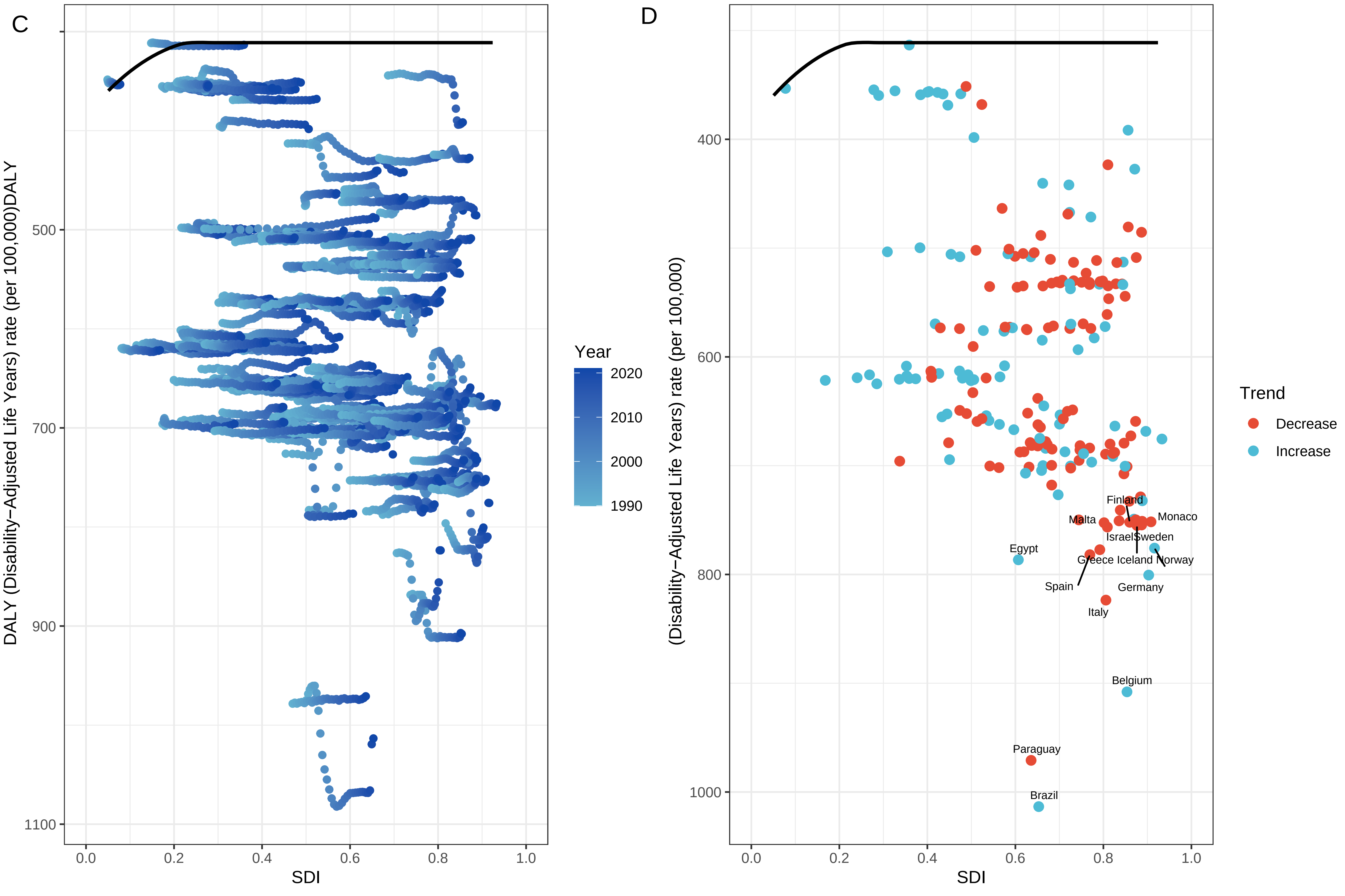

Supplementary figure 4: Frontier analysis based on SDI and DALY of migraine in AYA in 204 countries and territories:

(A) Frontier analysis based on SDI and 10-24 years migraine DALY rate from 1990 to 2021; (B) Frontier analysis based on SDI and 10-24 years migraine DALY rate in 2021; (C) Frontier analysis based on SDI and age-standardised migraine DALY rate from 1990 to 2021; (D) Frontier analysis based on SDI and age-standardised migraine DALY rate in 2021. (A), (C): Color scale represents the years from 1990 depicted in blue to 2021 depicted in gray. Solid black color to delineate the frontier. (B), (D): Dots represent countries and territories. The frontier is delineated in solid black color. Black fonts are used to label the top 15 countries with the largest effective difference (largest IBD DALY gap from the frontier). Examples of countries and territories with high SDI (>0.85) and relatively high effective difference for their level of development are labeled in red, and blue fonts are used to label examples of frontier countries with low SDI (<0.5) and low effective difference. Red dots indicate a decrease in 10-24 years or age-standardised migraine DALY rate from 1990 to 2021; blue dots indicate an increase in 10-24 years or age-standardised migraine DALY rate from 1990 to 2021.
