# Supplementary material for: Rising global burden of migraine among adolescents and young adults: a 30-year analysis (1990–2021)
Source: Front Neurol. 2025 Sep 1;16:1652468. doi: 10.3389/fneur.2025.1652468 (PMC12434965; doi:10.3389/fneur.2025.1652468)
Supplement: Supplementary file 6 [file Table_2.docx]

**Additional table 2: Female-to-Male Ratios of ASR for migraine in 1990 and 2021.**

|  | **ASIR** | | **ASPR** | | **ASDR** | |
| --- | --- | --- | --- | --- | --- | --- |
|  | **1990** | **2021** | **1990** | **2021** | **1990** | **2021** |
| Global | 1.66 | 1.59 | 1.69 | 1.62 | 1.68 | 1.61 |
| SDI Categories |  |  |  |  |  |  |
| High SDI | 2.11 | 2.09 | 2.00 | 1.98 | 1.99 | 1.96 |
| High-middle SDI | 1.78 | 1.76 | 1.77 | 1.73 | 1.75 | 1.71 |
| Middle SDI | 1.63 | 1.58 | 1.68 | 1.63 | 1.66 | 1.61 |
| Low-middle SDI | 1.49 | 1.47 | 1.57 | 1.54 | 1.56 | 1.53 |
| Low SDI | 1.46 | 1.46 | 1.50 | 1.49 | 1.49 | 1.48 |
| GBD Regions |  |  |  |  |  |  |
| High-income North America | 2.31 | 2.28 | 2.19 | 2.21 | 2.19 | 2.19 |
| Western Europe | 2.09 | 2.09 | 2.00 | 1.99 | 2.05 | 2.02 |
| High-income Asia Pacific | 2.14 | 2.05 | 1.95 | 1.94 | 1.92 | 1.91 |
| Southern Latin America | 1.95 | 1.95 | 1.99 | 1.89 | 1.91 | 1.87 |
| Eastern Europe | 1.87 | 1.86 | 1.91 | 1.88 | 1.87 | 1.86 |
| Central Latin America | 1.88 | 1.85 | 1.88 | 1.87 | 1.84 | 1.83 |
| Central Europe | 1.85 | 1.85 | 1.84 | 1.83 | 1.91 | 1.82 |
| Andean Latin America | 1.85 | 1.84 | 1.81 | 1.81 | 1.79 | 1.79 |
| Central Asia | 1.77 | 1.77 | 1.81 | 1.81 | 1.75 | 1.75 |
| Australasia | 1.71 | 1.71 | 1.78 | 1.78 | 1.75 | 1.75 |
| Caribbean | 1.70 | 1.70 | 1.78 | 1.71 | 1.76 | 1.68 |
| East Asia | 1.71 | 1.67 | 1.74 | 1.68 | 1.69 | 1.65 |
| North Africa and Middle East | 1.63 | 1.63 | 1.59 | 1.59 | 1.58 | 1.58 |
| Tropical Latin America | 1.63 | 1.59 | 1.60 | 1.56 | 1.62 | 1.56 |
| Eastern Sub-Saharan Africa | 1.54 | 1.53 | 1.56 | 1.53 | 1.55 | 1.52 |
| Southeast Asia | 1.54 | 1.52 | 1.52 | 1.52 | 1.52 | 1.51 |
| Central Sub-Saharan Africa | 1.46 | 1.46 | 1.51 | 1.51 | 1.51 | 1.51 |
| Oceania | 1.46 | 1.46 | 1.49 | 1.49 | 1.47 | 1.47 |
| Southern Sub-Saharan Africa | 1.45 | 1.45 | 1.48 | 1.48 | 1.47 | 1.46 |
| Western Sub-Saharan Africa | 1.42 | 1.43 | 1.46 | 1.47 | 1.45 | 1.45 |
| South Asia | 1.44 | 1.42 | 1.46 | 1.46 | 1.44 | 1.45 |
