# Supplementary material for: Rising global burden of migraine among adolescents and young adults: a 30-year analysis (1990–2021)
Source: Front Neurol. 2025 Sep 1;16:1652468. doi: 10.3389/fneur.2025.1652468 (PMC12434965; doi:10.3389/fneur.2025.1652468)
Supplement: Supplementary file 7 [file Table_3.docx]

|  | **Incidence rate** | | **Prevalence rate** | | **DALY rate** | |
| --- | --- | --- | --- | --- | --- | --- |
|  | **Female** | **Male** | **Female** | **Male** | **Female** | **Male** |
| Global | -0.21 | 4.02 | 0.86 | 5.23 | 0.88 | 5.3 |
| SDI Categories | | | | | | |
| High SDI | 3.93 | 5.04 | 1.76 | 3.21 | 1.23 | 3.08 |
| High-middle SDI | 4.51 | 5.92 | 3.05 | 5.58 | 3.18 | 5.53 |
| Middle SDI | 3.96 | 6.88 | 5.44 | 8.7 | 5.48 | 8.48 |
| Low-middle SDI | -3.24 | -1.92 | -0.35 | 1.73 | 0.12 | 2.41 |
| Low SDI | -0.65 | -0.95 | 0.09 | 0.75 | 0.93 | 1.81 |
| GBD Regions | | | | | | |
| East Asia | 8.32 | 10.3 | 6.23 | 7 | 6.5 | 7.33 |
| Andean Latin America | 2.81 | 2.46 | 4.32 | 4.7 | 2.53 | 7.32 |
| High-income Asia Pacific | -2.04 | 2.23 | 3.32 | 2.05 | 2.18 | 4.76 |
| Western Europe | 1.64 | 2.19 | 2.95 | 7.51 | 3.86 | 4.28 |
| High-income North  America | 0.43 | 2.03 | 1.95 | 5.34 | 1.64 | 3.48 |
| Eastern Europe | 1.1 | 1.75 | 1.68 | 3.22 | 2.45 | 3.1 |
| Southern Latin America | 1.52 | 1.4 | 1.65 | 2.37 | 0.94 | 2.69 |
| Australasia | 0.28 | 0.65 | 0.99 | 0.85 | -2.14 | 2.65 |
| Central Sub-Saharan  Africa | 0.14 | 0.2 | 0.5 | 0.79 | -1.14 | 2.46 |
| Central Asia | 0.19 | 0.18 | 0.32 | 0.74 | 3.14 | 2.27 |
| Eastern Sub-Saharan  Africa | 0.25 | 0.14 | 0.31 | 2.13 | 0.93 | 1.28 |
| Caribbean | 0 | -0.3 | -0.04 | 0.03 | 0.75 | 1.04 |
| Western Sub-Saharan  Africa | -0.18 | -0.56 | -0.12 | -0.15 | 0.95 | 0.97 |
| Oceania | -0.42 | -0.57 | -0.18 | -0.24 | 0.23 | 0.69 |
| Central Europe | -0.43 | -0.76 | -1.06 | -1.13 | -0.26 | 0.23 |
| Southern Sub-Saharan Africa | -0.49 | -0.79 | -1.09 | -0.7 | -0.14 | 0.03 |
| South Asia | -3.02 | -1.2 | -1.25 | -0.76 | -0.32 | -0.07 |
| Southeast Asia | -2.38 | -1.21 | -1.37 | 1.92 | -0.84 | -0.43 |
| North Africa and Middle East | -0.32 | -1.57 | -1.79 | -0.9 | -1.51 | -0.66 |
| Central Latin America | -2.42 | -1.63 | -2.31 | 2.82 | -2.27 | -0.88 |
| Tropical Latin America | -5.49 | -3.15 | -2.43 | -2.86 | -3.83 | -3.59 |

**Additional table 3: Percentage change rate by sex from 1990 to 2021.**
